# GOLPH3 protein controls organ growth by interacting with TOR signaling proteins in Drosophila

**DOI:** 10.1038/s41419-022-05438-9

**Published:** 2022-11-27

**Authors:** Anna Frappaolo, Angela Karimpour-Ghahnavieh, Giuliana Cesare, Stefano Sechi, Roberta Fraschini, Thomas Vaccari, Maria Grazia Giansanti

**Affiliations:** 1 Istituto di Biologia e Patologia Molecolari del CNR, c/o Dipartimento di Biologia e Biotecnologie, Sapienza Università di Roma, 00185 Roma, Italy; 2 Dipartimento di Bioscienze, Università degli Studi di Milano, 20133 Milano, Italy; 3 Dipartimento di Biotecnologie e Bioscienze, Università degli studi di Milano Bicocca, 20126 Milano, Italy

**Keywords:** TOR signalling, Cancer models

## Abstract

The oncoprotein GOLPH3 (Golgi phosphoprotein 3) is an evolutionarily conserved phosphatidylinositol 4-phosphate effector, mainly localized to the Golgi apparatus, where it supports organelle architecture and vesicular trafficking. Overexpression of human GOLPH3 correlates with poor prognosis in several cancer types and is associated with enhanced signaling downstream of mTOR (mechanistic target of rapamycin). However, the molecular link between GOLPH3 and mTOR remains elusive. Studies in *Drosophila melanogaster* have shown that Translationally controlled tumor protein (Tctp) and 14-3-3 proteins are required for organ growth by supporting the function of the small GTPase Ras homolog enriched in the brain (Rheb) during mTORC1 (mTOR complex 1) signaling. Here we demonstrate that *Drosophila* GOLPH3 (dGOLPH3) physically interacts with Tctp and 14-3-3ζ. RNAi-mediated knockdown of dGOLPH3 reduces wing and eye size and enhances the phenotypes of *Tctp* RNAi. This phenotype is partially rescued by overexpression of Tctp, 14-3-3ζ, or Rheb. We also show that the Golgi localization of Rheb in *Drosophila* cells depends on dGOLPH3. Consistent with dGOLPH3 involvement in Rheb-mediated mTORC1 activation, depletion of dGOLPH3 also reduces levels of phosphorylated ribosomal S6 kinase, a downstream target of mTORC1. Finally, the autophagy flux and the expression of autophagic transcription factors of the TFEB family, which anti correlates with mTOR signaling, are compromised upon reduction of dGOLPH3. Overall, our data provide the first in vivo demonstration that GOLPH3 regulates organ growth by directly associating with mTOR signaling proteins.

## Introduction

Mechanistic target of rapamycin (mTOR) is an evolutionarily conserved serine/threonine protein kinase, which controls cellular growth, proliferation, metabolism and survival as a core component of two multiprotein complexes termed mTORC1 and mTORC2 [1, 2]. The two complexes share the catalytic mTOR subunit and LST8 (lethal with SEC13 protein 8) [3, 4]. Specific components of mTORC1 are the regulatory-associated protein of mTOR (Raptor) [5, 6], the proline-rich Akt substrate 40 kDa (PRAS40) [7–9] and DEPTOR (DEP domain containing mTOR interacting protein) [10]. mTORC1 phosphorylates substrates that promote synthesis of proteins, lipids, nucleotides and ATP while suppressing catabolic autophagy [1]. mTORC1-mediated phosphorylation inhibits eukaryotic translation initiation factor 4E-binding protein (4E-BP) and activates p70 S6 kinase I (S6K1), driving protein translation of numerous proteins that regulate cell growth and differentiation [11, 12]. The small GTPase protein Rheb (Ras homolog enriched in brain) is an essential activator of mTORC1 in its GTP bound state [13–17], while the heterotrimeric tuberous sclerosis complex (TSC), negatively regulates the mTORC1 pathway by functioning as a GTPase-activating protein (GAP) and a negative regulator of Rheb [18–20]. Growth factors like insulin/insulin-like growth factor-1 activate mTORC1 signaling, through Akt kinase activation, which in turn phosphorylates the TSC2 component of the TSC complex, thus inhibiting the GAP activity of the TSC complex and relieving Rheb repression [13, 21].

The conserved Translationally controlled tumor protein (Tctp) protein has been also involved in mTOR signaling [22, 23]. Genetic analysis in *Drosophila* identified Tctp as a new molecular component in the TSC-Rheb pathway required for controlling organ growth by regulating Rheb GTPase activity [22, 24]. The *Drosophila* growth defects associated with Tctp knockdown are suppressed by expression of human Tctp, suggesting that the role of Tctp in growth regulation is conserved in human cells. Studies in mammals and *Drosophila* have shown that 14-3-3 proteins participate in mTORC1 signaling [24, 25]. In *Drosophila*, single knockdown of either 14-3-3ε or 14-3-3ζ did not result in defects in wing and eye growth but it strongly enhanced the *Tctp* RNA interference (RNAi) phenotypes indicating genetic interaction. 14-3-3ε and 14-3-3ζ proteins are binding partners of Tctp and Rheb proteins and regulate Tctp/Rheb interaction [24].

Recent work in a variety of mammalian cells has located several components of the mTORC1 pathway to the Golgi apparatus and indicated that it could be directly involved in mTORC1 activation [26–31]. Golgi phosphoprotein 3 (GOLPH3) is a highly conserved phosphatidylinositol 4-phosphate [PI(4)P] binding protein, mainly localized to the trans-Golgi network and required for multiple cellular processes in quiescent and dividing cells [32–34]. We have previously demonstrated that the *Drosophila* GOLPH3 (dGOLPH3) protein, encoded by the *sauron (sau)* locus, accumulates at the cleavage site during telophase and acts as a key molecule during cytokinesis to couple PI(4)P signaling and membrane remodeling with actomyosin ring dynamics [35–37]. Frequent overexpression of GOLPH3 has been correlated with poor prognosis in multiple cancer types including breast cancer, colon cancer and glioblastoma [33, 38, 39]. The oncogenic activity of human GOLPH3 has been correlated with enhanced activity of growth factor-induced mTOR signaling [39]. However, the molecular link between GOLPH3 with the mTOR-pathway remains elusive [33].

In this paper, we demonstrate that dGOLPH3 is required for organ growth by interacting with the Akt/mTOR signaling. We present biochemical data indicating that dGOLPH3 directly binds Lst8, the shared subunit of mTORC1/mTORC2 complexes. Moreover, dGOLPH3 physically interacts with Tctp and 14-3-3ζ proteins. Wing- and eye-specific RNAi-mediated knockdown of dGOLPH3 reduces the organ size, mimicking the effects of mutations in *Tctp*. Such reduction is partially rescued by overexpression of Tctp, 14-3-3ζ or Rheb. We show that localization of Rheb protein on the Golgi stacks in *Drosophila* cells depends on dGOLPH3. Consistent with the involvement in Rheb-mediated mTORC1 activation, depletion of dGOLPH3 reduces the level of phosphorylated S6K. Because mTOR signaling is intimately connected with regulation of autophagy by transcription factors of the TFEB family (Mitf in *Drosophila*) [40–42], we also investigated autophagic activity upon modulation of dGOLPH3. We have found that in *dGOLPH3* mutants or upon *dGOLPH3* depletion, the autophagy flux is decreased and expression of autophagic Mitf target genes is reduced. Overall, these data provide the first in vivo demonstration that GOLPH3 regulates organ growth by directly associating with the TOR signaling proteins.

## Results

### dGOLPH3 physically interacts with Lst8 and Tctp proteins

**Fig. 1:**
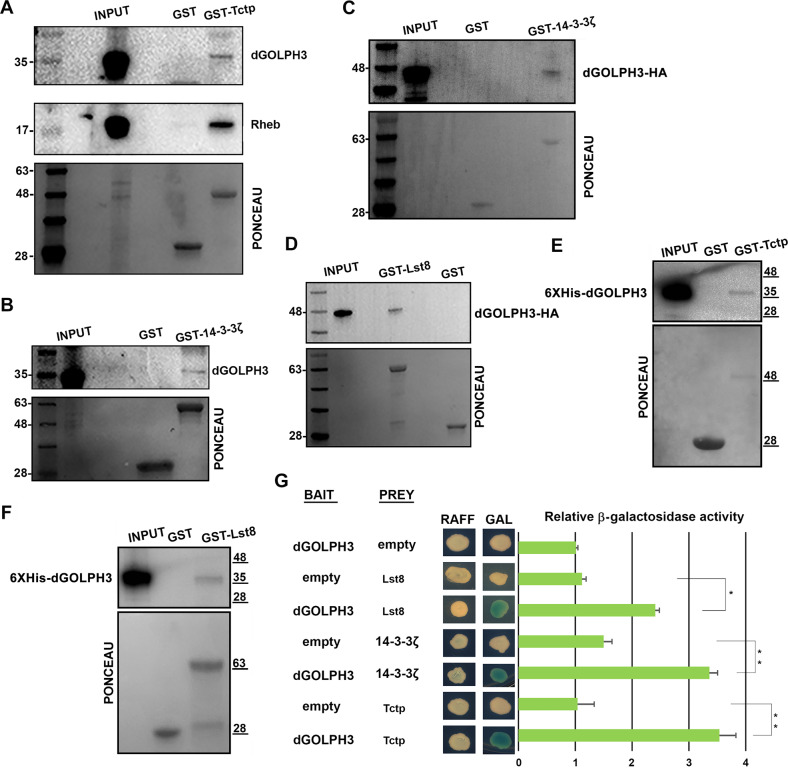
dGOLPH3 protein interacts with TOR signaling proteins. **A**, **B** GST–Tctp (**A**) and GST-14-3-3ζ (**B**) but not GST precipitated dGOLPH3 from protein extracts of S2 cells. GST-Tctp also precipitated Rheb protein. **C**, **D** GST-14-3-3ζ (**C**) and GST–Lst8 (**D**) but not GST precipitated dGOLPH3-HA protein from testis extracts. See Fig. S1A for the expression of dGOLPH3-HA. **A**–**D** Experiments were performed three times with identical results. Ponceau staining is shown as a loading control. 2% of the input and 25% of the pull-downs were loaded and probed for the indicated proteins. **E**, **F** 6XHis-dGOLPH3 directly binds GST-Tctp and GST-Lst8 proteins. Experiments were performed three times with identical results. Ponceau staining is shown as a loading control. 2% of the input and 25% of the pull-downs were loaded and probed for the indicated protein. (**A**–**F**) Molecular mass in kilodaltons. **F** Yeast two-hybrid assay was used to test dGOLPH3 interaction with Tctp, Lst8, and 14-3-3ζ. In the presence of the dGOLPH3 bait, all the indicated proteins induce LacZ expression (blue color indicates positive interaction). Graph shows quantification of LacZ reporter expression induced with different combinations of bait and prey plasmids. Error bars, SEM; *P < 0.05, **P < 0.01 (unpaired *t*-test), *n* = 3.

By affinity purification coupled with mass spectrometry (AP-MS) using dGOLPH3-RFP expressed in *Drosophila* testes as a bait, we identified several interacting proteins involved in mTOR signaling [34]. To further characterize such interactions, we carried out glutathione S-transferase (GST) pull-down experiments using either extracts of *Drosophila* S2 cells or testis extracts expressing hemagglutinin tagged dGOLPH3 (dGOLPH3-HA; Fig. S1A). In agreement with previous studies that indicated direct association of Rheb with Tctp [24], GST-Tctp pulled down Rheb (Fig. 1A), while dGOLPH3 and dGOLPH3-HA precipitated with GST-Tctp, GST-14-3-3ζ and GST-Lst8 (Figs. 1A–D and S1A). Furthermore, GST pull-down assays, using bacterially expressed fusion proteins, demonstrated direct binding of Tctp and Lst8 to dGOLPH3 (Fig. 1E, F). Direct interaction of dGOLPH3 with Tctp, 14-3-3ζ and Lst8 was confirmed by using yeast two-hybrid assays (Fig. 1G). These data show the capacity of GOLPH3 to bind Lst8, 14-3-3ζ and Tctp proteins.

### *Drosophila dGOLPH3/sau* controls organ growth

**Fig. 2:**
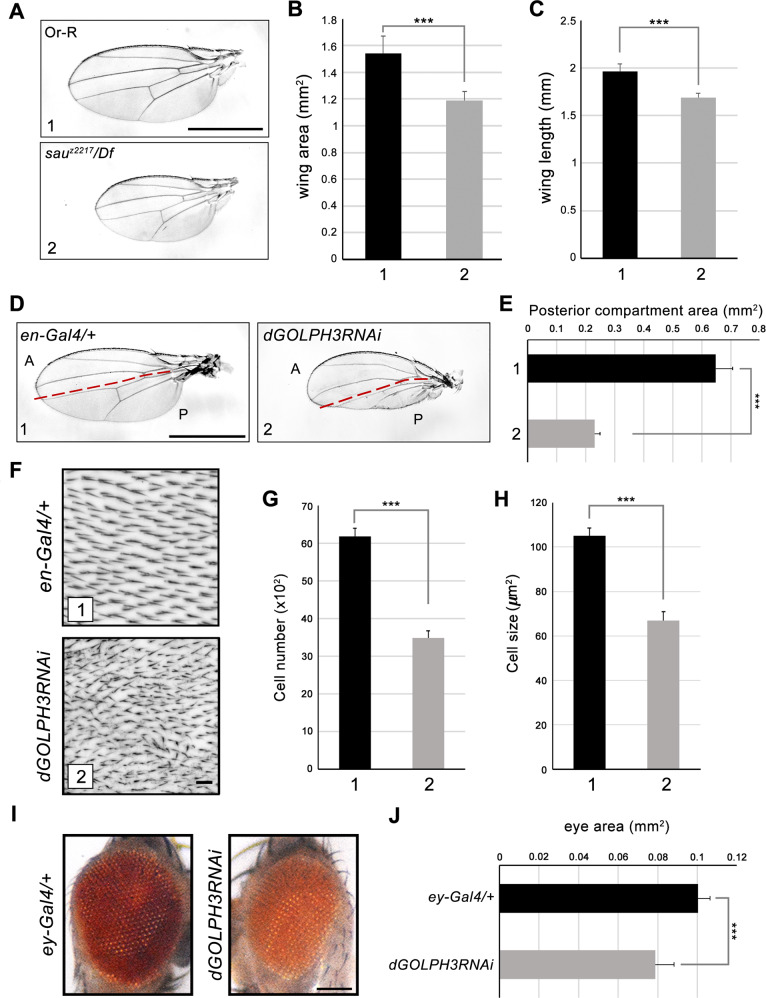
Flies carrying *sau*^*z2217*^ mutation or depleted of dGOLPH3 exhibit organ growth defects. **A** *sau*^*z2217*^*/Df(2* *L)Exel7010* (*sau*^*z2217*^*/Df*) mutant flies exhibit small wings compared to the wild type (Or-R). Scale bar, 1 mm. **B**, **C** Quantification of wing sizes shown in **A**. Error bars indicate SD, *n* = 10. ***P < 0.0001(unpaired *t*-test). **D** Knockdown of dGOLPH3 by means of *en-Gal4* (*dGOLPH3*RNAi) results in the reduction of the posterior compartment of the wing compared to the control (*en-Gal4/+*). Red dashed lines mark the anterior (A)/posterior (P) boundary. Scale bar, 1 mm. **E** Quantification of the posterior compartment area in the wings shown in **D**. Error bars, SD; *n* = 15. ***P < 0.0001 (unpaired *t*-test). **F** Higher magnification images for the area of 22,500 μm^2^ selected next to the posterior crossvein in the wing compartment shown in **D**. Scale bar, 10 μm. **G**, **H** Quantification of the total number of cells (**G**) and of the cell size (**H**) from images shown in **F**. The small wing phenotype shown in **D** is due to smaller cell size (about 35% decrease) and decrease in cell number (~45% decrease). Error bars, SD; *n* = 15. ***P < 0.0001(unpaired *t*-test). **I** Knockdown of dGOLPH3 by means of *ey-Gal4* affects the eye size. Flies carrying one copy of *ey-Gal4 (ey-Gal4/+)* show normal eye; *ey-Gal4* > *dGOLPH3*RNAi (*dGOLPH3RNAi*) flies display small and rough eye. Scale bar, 0.1 mm. **J** Quantification of the eye area (*n* = 10), measured from images shown in **I**. Error bars, SD; ***P < 0.0001 (unpaired *t*-test). See the related Fig. S1B.

To investigate a possible role of GOLPH3 in mTOR signaling and tissue growth, we analyzed the effect of impairment of *dGOLPH3*/*sau* in vivo. Remarkably, the wings of *sau*^*z2217*^*/Df(2* *L)Exel7010* mutant flies, which carry the missense allele of *dGOLPH3 sau*^*z2217*^ [35], displayed a significant reduction in size, when compared to control flies (Fig. 2A–C), a phenotype reminiscent of reduced mTOR signaling. Next, we used the Gal4/UAS binary system [43] to reduce *dGOLPH3* expression tissue specifically by RNA interference (RNAi). As shown previously [35], ubiquitous in vivo expression of *dGOLPH3* RNAi [44] efficiently reduced endogenous dGOLPH3 protein levels (Fig. S1B). Consistent with the reduction observed in *sau* mutants, dGOLPH3 depletion driven by *engrailed-Gal4 (en-Gal4)*, which is expressed in the posterior compartment of wing imaginal disks (*en* > *dGOLPH3*), resulted in reduction of the posterior part of the adult wing (Fig. 2D, E). To analyze the effects of *en* > *dGOLPH3* RNAi on cell number and size, we examined the hair wing density. Knockdown of dGOLPH3 increased the wing hair density in the posterior compartment suggesting a decrease in cell number and size (Fig. 2F–H). We next tested whether silencing of *dGOLPH3* causes similar growth defects in the eye. dGOLPH3 depletion (*ey* > *dGOLPH3* RNAi) using *ey-Gal4*, that drives expression in the eye and head primordia, leads to strong reduction of the eye size (Fig. 2I, J). Overall, these results suggest that dGOLPH3 is required to control tissue and organ growth during fly development.

### *Drosophila Tctp*, *Rheb*, and *14-3-3*ζ interact genetically with *dGOLPH3*

**Fig. 3:**
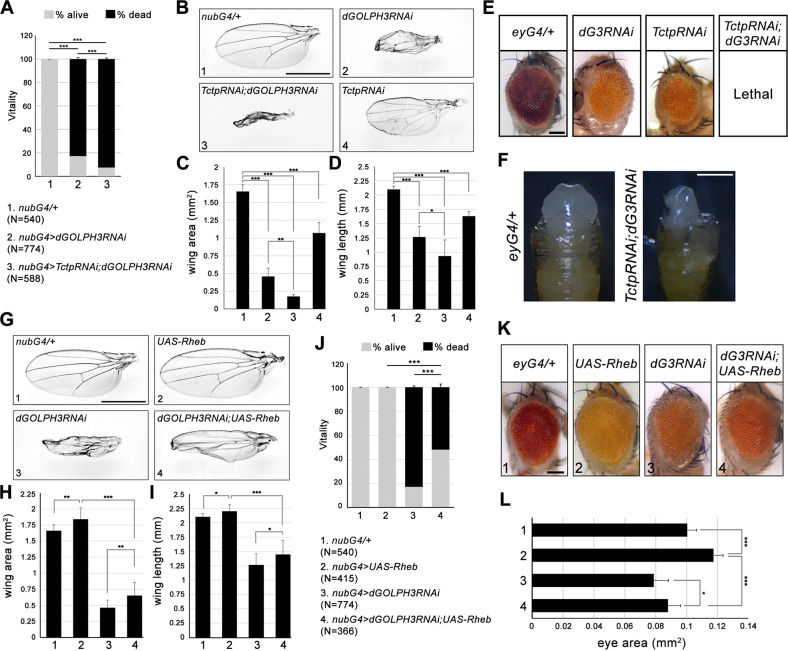
*Tctp* and *Rheb* genetically interact with *dGOLPH3*. **A** Percentage of flies emerging from the pupal case. N, total number of pupae. *nub-Gal4/+* (*nubG4/+*, 1), wild type. Double knockdown of Tctp and dGOLPH3 results in a significant increase of lethality. Error bars, SD; ***P < 0.0001 (Fisher’s exact test). **B** Genetic interaction between *Tctp* and *dGOLPH3* in the wing. *nub-Gal4/+* (*nubG4/+*, 1) wild type wing; *dGOLPH3* RNAi (2) leads to severe size reduction and wrinkling of wings; *Tctp* RNAi (4) causes reduction and mild wrinkling of wings; knockdown of Tctp strongly enhances the effects of *dGOLPH3* RNAi (3) phenotype. Scale bar, 1 mm. **C**, **D** Quantification of wing sizes shown in **B**. *dGOLPH3* RNAi (2) reduces the wing area about 70% and the wing length about 40% compared with the wild-type; *dGOLPH3* RNAi combined with *Tctp* RNAi (3), reduces the wing area about 60% and the wing length about 10% compared with *dGOLPH3* RNAi. Error bars, SD; *n* = 15. *P < 0.05; **P < 0.01; ***P < 0.0001 (unpaired *t*-test). **E** Genetic interaction between *Tctp* and *dGOLPH3* in the eye. *ey-Gal4/+*, wild-type eye (*eyG4/+*). Knockdown of either Tctp or dGOLPH3 (*dG3RNAi*) results in small and rough eyes. Scale bar, 0.1 mm. **F** Double knockdown of both Tctp and dGOLPH3 causes loss of targeted tissues in the pupae. Scale bar, 0.5 mm. **G** Genetic interaction between *Rheb* and *dGOLPH3* in the wing. *nub-Gal4/+* (*nubG4/+*, 1), wild-type wing; Rheb overexpression (2), *dGOLPH3* RNAi (3), *dGOLPH3* RNAi combined with Rheb overexpression (4). Scale bar, 1 mm. **H**, **I** Quantification of wing sizes in animals shown in G. Error bars, SD; *n* = 15. *P < 0.05; **P < 0.01; ***P < 0.0001 (unpaired t-test). **J** Percentage of flies emerging from the pupal case. N, total number of pupae. *nubG4* > *dGOLPH3* RNAi combined with *Rheb* overexpression (4), leads to 52% of pupal lethality. Error bars, SD; ***P < 0.0001 (Fisher’s exact test). **K** Genetic interaction between *Rheb* and *dGOLPH3* in the eye. *ey-Gal4/+*, wild type (*eyG4/+*). Overexpression of Rheb increases the eye size. Rheb overexpression partially suppresses the effects of *dGOLPH3* RNAi (*dG3RNAi*). Scale bar, 0.1 mm. **L** Quantification of eye sizes shown in **K**. Error bars, SD; *n* = 10. *P < 0.05; ***P < 0.0001 (unpaired t-test). See also the associated Fig. S1C, D.

To test whether the activity of dGOLPH3 in promoting growth is mediated by the mTOR pathway, we assessed genetic interaction between *dGOLPH3* and *Tctp*. To this end, we depleted *dGOLPH3* in developing wing disks using *nubbin-Gal4* (*nub* > *dGOLPH3* RNAi). Upon dGOLPH3 depletion, most of the progeny dies during the late pupal stage (Fig. 3A). However, flies surviving to adulthood displayed wings of reduced size, similar to efficient depletion of *Tctp* (Figs. S1C and 3B–D). Interestingly, co-depletion of *dGOLPH3* and *Tctp* significantly enhanced wing size reduction (Fig. 3B–D). The genetic interaction between *Tctp* and *dGOLPH3* was confirmed in the adult compound eye using *eyless-Gal4*-driven depletion (*ey*>). In agreement with previous studies, *ey* > *Tctp* RNAi resulted in small and rough eyes, while double knockdown of dGOLPH3 and Tctp resulted in pupal lethality (Fig. 3E). However, removal of the pupal case from dead pupae of *ey* > *Tctp* RNAi, *dGOLPH3* RNAi revealed complete loss of eye-head structures (Fig. 3F).

We also examined whether the *dGOLPH3* might also interact genetically with *Rheb*. To this end, we first assessed that Rheb could be efficiently overexpressed (Fig. S1D). Consistent with previous studies [24], *nub-Gal4*-mediated overexpression of Rheb induced 10% increase in the wing area and 5% increase in the wing length, when compared with control (Fig. 3G–I). Interestingly, Rheb overexpression partially rescued the small-wing size phenotype resulting from *dGOLPH3* RNAi (Fig. 3G–I). In addition, Rheb overexpression combined with *dGOLPH3* RNAi resulted in a decrease in pupal lethality, when compared to *dGOLPH3* RNAi alone (Fig. 3J). Moreover, Rheb overexpression partially suppressed the eye size defects associated with knockdown of dGOLPH3 (Fig. 3K, L).

**Fig. 4:**
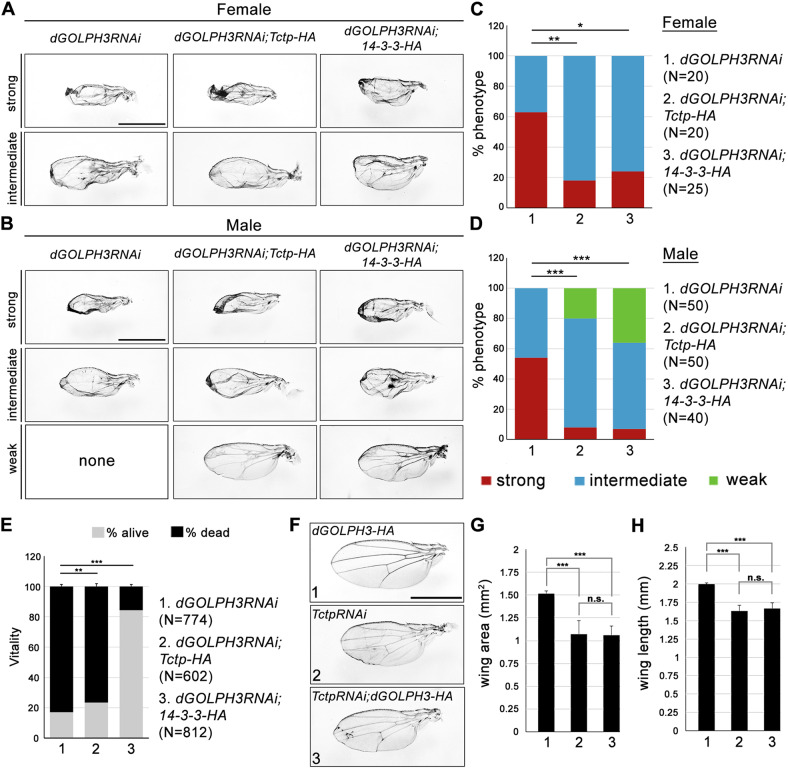
Overexpression of either Tctp or 14-3-3ζ proteins, can partially suppress the *dGOLPH3 RNAi* phenotype. **A**, **B** Wing phenotypes in females (**A**) and males (**B**) from *nub* > *dGOLPH3* RNAi flies and *nub* > *dGOLPH3 RNAi* flies expressing either Tctp-HA or 14-3-3ζ-HA (14-3-3-HA). Weak, mild reduction of the wing size; intermediate, wing size reduction and mild folding; strong, showing consistent reduction with severe folding. Scale bars in **A**, **B**, 1 mm. See the associated Fig. S1E. **C**, **D** Quantification of rescue shown in **A**, **B**. N, number of wings. *P < 0.05; **P < 0.01; ***P < 0.0001 (Fisher’s exact test). **E** Percentage of flies emerging from the pupal case. N, total number of pupae. Error bars, SD; **P < 0.01; ***P < 0.0001 (Fisher’s exact test). **F** Overexpression of GOLPH3-HA protein does not suppress the *Tctp* RNAi wing phenotype. Scale bars, 1 mm. **G**, **H** Quantification analysis of wing sizes from animals shown in **F**. Error bars, SD; *n* = 15. ***P < 0.0001; n.s., not significant: *p* = 0.8214 in G; *p* = 0.4013 in H (unpaired *t*-test).

We finally tested whether growth defects associated with *dGOLPH3* depletion could be suppressed by efficient overexpression of either Tctp-HA or 14-3-3ζ-HA (Figs. 4A–E and S1E). We observed that overexpression of either Tctp-HA or 14-3-3ζ-HA partially rescued the pupal lethality and the wing size defects associated with *dGOLPH3* RNAi (Fig. 4A–E). In contrast, overexpression of dGOLPH3-HA did not rescue the small-wing phenotype associated with Tctp depletion (Fig. 4F–H) indicating that Tctp and 14-3-3ζ, are downstream effectors of dGOLPH3. In sum, these data suggest that dGOLPH3 is a functional component of the mTOR growth control machinery, acting genetically upstream of Tctp and 14-3-3ζ.

### dGOLPH3 promotes Golgi localization of Rheb and Rheb interaction with Tctp

**Fig. 5:**
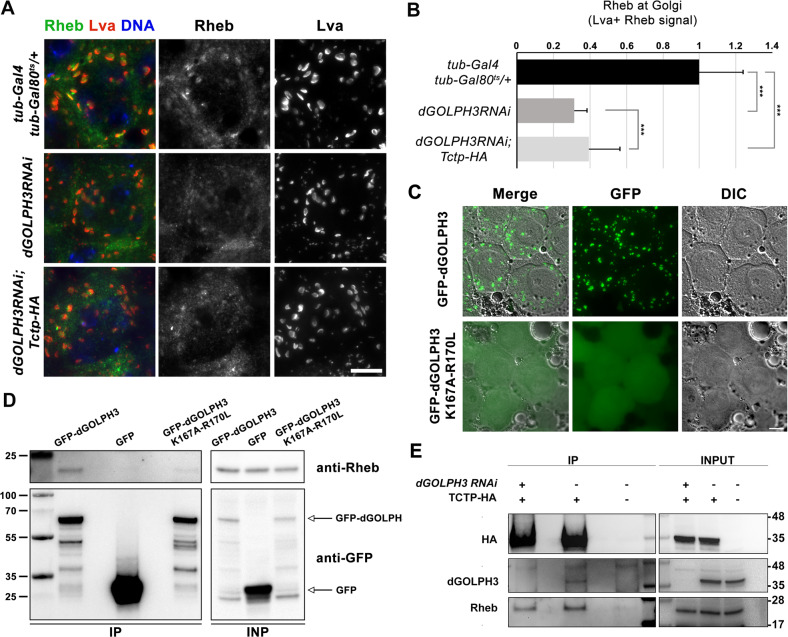
dGOLPH3 is required for Rheb recruitment to the Golgi and to promote Rheb interaction with Tctp. **A** *Drosophila* spermatocytes stained for Rheb (green), Lva (red), and DNA (blue). Scale bar, 10 μm. The following genotypes were used: *tub-Gal4 tub-Gal80*^*ts*^ */+* as a control; *tub-Gal4 tub-Gal80*^*ts*^ > *dGOLPH3RNAi* (*dGOLPH3RNAi*) and *tub-Gal4 tub-Gal80*^*ts*^ > *dGOLPH3RNAi* overexpressing Tctp-HA (*dGOLPH3RNAi*;*Tctp-HA*). **B** Rheb levels at the Golgi have been quantified as mean fluorescence intensity in Lva positive (Lva^+^) regions (see material and methods for further details). Error bars, SD; ***P < 0.0001(unpaired *t*-test). *n* = 100, number of Golgi stacks analyzed and randomly selected from three independent experiments. **C** Fluorescence and corresponding DIC micrographs of live wild type spermatocytes expressing either GFP-dGOLPH3 or GFP-dGOLPH3^K167A-R170L^ during prophase. Note that GFP- dGOLPH3^K167A-R170L^ accumulates in the cytoplasm. Scale bar, 10 μm. **D** Co-IP analysis from protein extracts of pupae expressing GFP, GFP-dGOLPH3 or GFP-dGOLPH3^K167A-R170L^. Rheb protein co-immunoprecipitates with GFP-dGOLPH3 but not with GFP or GFP-dGOLPH3^K167A-R170L^. 2% of the input and 50% of the immunoprecipitates were loaded and probed with anti-Rheb and anti-GFP antibodies. Molecular masses are in kilodaltons. Experiments were performed three times with identical results. Related to Fig. S2. **E** dGOLPH3 and Rheb proteins co-immunoprecipitate with Tctp-HA from protein extracts of *Drosophila* pupae. Note that the interaction between Tctp-HA and Rheb was subtly reduced by knockdown of dGOLPH3. 2% of the input and 25% of the immunoprecipitates were loaded and probed with the indicated antibody. Molecular masses are in kilodaltons. Experiments were performed three times with identical results. Related to Fig. S3.

How could dGOLPH3 modulate the activity of mTOR complex components? One possibility is that dGOLPH3 might promote regulation of mTORC1 at the Golgi apparatus. Because recent data reported localization of human Rheb to the Golgi apparatus [28, 30, 31], we examined whether Rheb localizes to Golgi stacks. For this analysis we used *Drosophila* spermatocytes, which offer a highly suitable cell system for the analysis of the Golgi apparatus [45]. Interestingly, we observed that Rheb localized to Golgi stacks of primary spermatocytes, as assessed by co-staining with the Golgi marker Lava lamp (Lva, [46]; Fig. 5A, quantified in B). Consistent with the possibility that dGOLPH3 might be required for Rheb association to the Golgi apparatus, dGOLPH3 depletion decreased the amount of Rheb protein localized to the Golgi apparatus (Fig. 5A, quantified in B). Next, we performed co-IP assays using extracts from tissues expressing either GFP-tagged dGOLPH3 or GFP-dGOLPH3^K167A-R170L^, a GFP-mutant version of dGOLPH3 carrying two substitutions in the PI(4)P binding pocket that impair localization to Golgi stacks ([35], Fig. 5C). Remarkably, Rheb co-precipitates with GFP-dGOLPH3 but not with GFP-dGOLPH3^K167A-R170L^ (Fig. 5D). Consistent with this, we found that Rheb immunoprecipitated also with RFP-tagged dGOLPH3 [34], from extracts of *Drosophila* pupae (Fig. S2A, B), indicating that dGOLPH3 is a molecular partner of Rheb in vivo. Taken together, these results suggest that dGOLPH3 might recruit Rheb to the Golgi apparatus. Since dGOLPH3 appears to bind both Tctp and Rheb, we finally tested whether dGOLPH3 could influence the ability of Tctp and Rheb to form a complex in animals overexpressing Tctp-HA. Co-IP analysis showed that dGOLPH3 co-immunoprecipitates with Tctp-HA further corroborating the association of dGOLPH3 with Tctp in a protein complex (Fig. 5E). Interestingly, knockdown of dGOLPH3 slightly reduced binding of the overexpressed Tctp-HA protein to Rheb, as assayed by Co-IP (Fig. 5E). Overall levels of Tctp and Rheb were not altered by depletion of dGOLPH3 (Figs. 5E and S3) indicating that reduced Tctp-Rheb binding was not the result of a decreased protein concentration. However, consistent with the Co-IP analysis, overexpression of Tctp-HA only partially rescued *dGOLPH3* RNAi -associated decrease in Rheb localization to the Golgi in primary spermatocytes (Fig. 5A, B). These data suggest that dGOLPH3 might regulate formation of Rheb/Tctp complexes by recruiting Rheb to the Golgi apparatus.

### dGOLPH3 is required for regulation of pathways downstream of mTOR

**Fig. 6:**
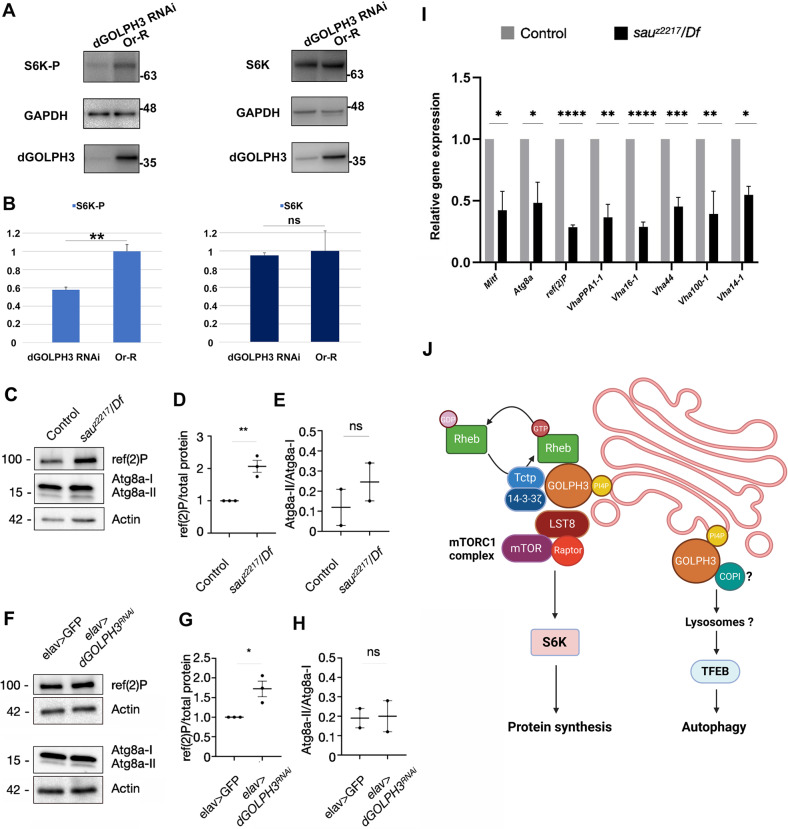
Roles of dGOLPH3 in S6k phosphorylation and autophagy. **A** Western blot from adult testis extracts of wild type (Or-R) and *Bam* > *dGOLPH3RNAi* (*dGOLPH3* RNAi) flies to test the effects of *dGOLPH3* RNAi on S6K protein phosphorylation. Knockdown of dGOLPH3 affects the level of pS6K (S6K-P) but does not reduce the level of S6K. GAPDH levels were used as loading controls. Molecular masses are in kilodaltons. **B** Quantification of the expression levels of S6K and pS6K (S6K-P) proteins in western blots from adult testis extracts. Band intensities are from three independent experiments. The intensity of each band relative to the intensity of loading control was normalized to the wild-type control (see material and methods for further details). Error bars, SD. **p < 0.001; n.s., not significant, *p* = 0.0597 (unpaired *t*-test). **C**–**H** Western blot analysis showing levels of the autophagy components ref(2)P and Atg8a in control and in *dGOLPH3* mutant (*sau*^*z2217*^*/Df*) head extracts (**C**, one of 3 experiments is shown), or in control (*elav* > *GFP*) and *dGOLPH3* knockdown (*elav* > *dGOLPH3RNAi*) head extracts (**F**, one of 3 experiments is shown). Actin was used as a loading control. Molecular masses are in kilodaltons. Blots indicate protein levels normalized for actin and represent the fold change relative to the loading control (**D**, **G**) or levels of the active form Atg8a-II relative to the levels of the inactive form Atg8a-I (**E**, **H**). The ref(2)P protein levels are increased in *dGOLPH3* mutants and upon dGOLPH3 depletion, whereas levels of the active form Atg8a-II are not significantly changed. **I** RT-qPCR analysis to assess gene expression levels of Mitf targets in 3 days old fly heads. Black bars show the relative mRNA levels of Mitf target genes in *dGOLPH3* mutant fly heads versus control fly heads, in gray. Gene expression was normalized relative to *Rp49* (*RpL32*) gene levels. Data shown are the mean of replicates (*n* = 3) ±SEM. Statistics analysis was performed on fold change. *P < 0.05; **P < 0.005, ***P < 0.0005, ****P < 0.0001 (unpaired *t* test). **J** Schematic (depicted with BioRender) of the role of dGOLPH3 in the mTORC1 signaling. mTORC1-mediated phosphorylation activates p70 S6 kinase I (S6K), driving protein synthesis. Rheb is an essential activator of mTOR in its GTP bound state. Previous work showed that Tctp forms a complex with 14-3-3 proteins and Rheb and acts as a GEF for Rheb. Our data suggest that PI(4)P-bound dGOLPH3 protein is required to target Rheb protein to the Golgi membranes and to facilitate Tctp-Rheb association. Our data also support a role for dGOLPH3 in the autophagy. Recent data indicate that inhibition of COPI vesicle biogenesis impairs lysosomal trafficking and activation of mTORC1. We may speculate that the dGOLPH3-COPI module functions in both intra-Golgi trafficking and lysosomal trafficking of mTORC1 with an impact on TFEB-mediated lysosomal signaling.

To assess whether dGOLPH3 regulates the activity of mTOR signaling effectors, we first tested whether activation of dS6K was altered upon dGOLPH3 depletion. Importantly, knockdown of dGOLPH3 in testes, resulted in a significant reduction of pS6K detected by anti-pS6K antibody, while the total level of S6k protein remained unchanged (Fig. 6A, B). Then, we investigated whether dGOLPH3 inactivation or depletion affects autophagy, which is coregulated with mTOR signaling [2, 27, 47, 48]. In vivo, in extracts from dGOLPH3 mutants or from flies depleted of dGOLPH3, we observed accumulation of ref(2)P, the fly homolog of the autophagy adapter p62 [49] while lipidation of the autophagosome protein Atg8a (LC3 in mammals) is not significantly affected (Fig. 6C–H). These data suggest that autophagy is reduced. We also analyzed expression levels of validated target genes of Mitf, the single fly homolog of the TFEB family [50, 51]. We found that expression of Mitf target genes is significantly reduced in *dGOLPH3* mutant flies, indicating that induction of autophagy is impaired (Fig. 6I). Thus, dGOLPH3 is required to control anabolism and catabolism regulated by mTOR signaling.

## Discussion

Our study provides the first compelling evidence that dGOLPH3 controls cell growth and cell number during organ development by directly affecting the Tctp-Rheb-mTORC1 axis. We suggest that dGOLPH3 is required to recruit Rheb to the Golgi apparatus and to promote formation of the Rheb-Tctp complex that supports activation of mTORC1 (Fig. 6J). Direct interaction of dGOLPH3 with Lst8, 14-3-3ζ and Tctp, indicates that more extensive portions of the mTOR signaling might operate at the Golgi apparatus, however it is not yet clear whether the all the interactions entertained by dGOLPH3 with mTOR components occur at the Golgi apparatus and how interactions result in activation of mTORC1.

In agreement with our findings, many reports have located several mTORC1 components as well as the immediate upstream activator Rheb to the Golgi apparatus, leading to propose that the Golgi complex provides a hub for mTOR activation [26–31].

It is known that localization of Rheb to membranes depends on farnesylation of the Rheb COOH-terminal CaaX (C is cysteine, A is any aliphatic residue, X can be variable amino acids) motif [26]. However, since Rheb has been found localized to a number of different endomembranes, it is not clear whether specific membrane targeting motifs mediate its binding to Golgi membranes [28, 52]. We find that a form of dGOLPH3 that cannot bind to Golgi membranes, fails to bind Rheb, suggesting that the interactions between the two might occur preferentially at the Golgi apparatus. Thus, dGOLPH3 might provide specificity to target Rheb to Golgi membranes (Fig. 6J). Once there, dGOLPH3 might facilitate Tctp-Rheb association, as indicated by our findings that depletion of dGOLPH3 does not significantly affect the protein levels of Rheb and Tctp, but rather reduces the efficiency of Tctp-Rheb complex formation. Rab1 GTPase, a small GTPase known to regulate ER to Golgi and intra-Golgi trafficking, has been also identified as a key activator of the mTORC1 pathway at the Golgi apparatus [31]. Similar to GOLPH3, human Rab1A functions as an oncogene in breast cancer [53], colorectal carcinoma [31] and hepatocellular carcinoma [54]. Overexpression of dGOLPH3 enhances mTORC1 signaling, promotes oncogenic transformation and growth, and correlates with tumor progression and poor prognosis in colorectal cancer patients [33, 39]. Mechanistically, it has been shown that Rab1A binds the mTORC1 subunit Raptor, controls Rheb-mTORC1 interaction in the Golgi and TORC1 activation [31]. Importantly, our previous work showed that dGOLPH3 binds and behaves as an effector of Rab1 in *Drosophila*, and these data have been confirmed for human GOLPH3 by another group [36, 55]. Combined, these findings suggest that GOLPH3 and Rab1 might act in the same pathway controlling Rheb-mediated activation of mTORC1 at the Golgi.

The interaction of dGOLPH3 with Lst8 posits that dGOLPH3 might also regulate mTORC2 activity. Although mLST8/Lst8 is a shared component of both mTORC1 and mTORC2, increasing evidence in *Drosophila* and mammalian cells, suggests that this protein is dispensable for mTORC1 activity but required for mTORC2 function [56, 57]. A recent study in a panel of normal and cancer cells showed that mLST8 acts as a scaffold protein for assembly and activity of mTORC2. Indeed, absence of mLST8 blocks selectively the association of mTOR with mTORC2 cofactors RICTOR and SIN1 [58]. In addition, we validated the interaction of dGOLPH3 with 14-3-3ζ protein that was identified in our previous analysis of dGOLPH3 interactome by affinity purification from testis extracts [34]. Previous studies have shown that both 14-3-3ε and 14-3-3ζ are molecular partners of Tctp and Rheb proteins and promote Tctp/Rheb interaction [24]. Further investigation will be required to clarify whether dGOLPH3 functions with Lst8 in mTORC2 signaling and whether 14-3-3ε contributes to regulation of Rheb activity. Consistent with the role of dGOLPH3 in Rheb-mediated mTORC1 activation, knockdown of dGOLPH3 causes a significant reduction of phosphorylated ribosomal S6 kinase, a downstream target of mTORC1. In addition, we find that dGOLPH3 inactivation reduces autophagy. This is an unexpected effect of reduced mTORC1 activity, as inactivation of mTOR signaling normally correlates with TFEB activation and induction of autophagy [40–42]. In addition, because GOLPH3 is required to support Golgi apparatus integrity and the latter has been reported to inhibit autophagy [27] we expected induction of autophagy upon inactivation of dGOLPH3. While GOLPH3 has been recently involved in regulation of autophagy in human cultured cells and in a few tumor contexts [38, 59, 60], its role in the process is not understood. Our findings indicating that autophagy is supported by dGOLPH3 at the step of transcriptional induction of autophagy regulated by Mitf/TEB, suggest that loss of dGOLPH3 might inhibit translocation of TFEB from lysosomes to the nucleus. As recent reports indicate that GOLPH3 regulates Golgi activities that impact on lysosomal biogenesis [61], we envisage that GOLPH3 might be required to enable lysosomal signaling by TFEB. In such scenario, the impairment of mTORC1 signaling observed upon loss of dGOLPH3 could be in part due to alteration of lysosome activity and association of mTOR factors, including Rheb, to lysosomes. However, whether defects in autophagy are a consequence of changes in lysosomal function, or whether they depend on the failure to activate mTORC1 at the Golgi apparatus remains to be determined. In this context, it has also been reported that inhibition of COPI vesicle biogenesis impairs lysosomal trafficking and activation of mTORC1 in mammalian cells [62] and that ARF1 is involved in glutamine-stimulated lysosomal mTORC1 localization and activation [63, 64]. Because GOLPH3 family proteins, including dGOLPH3, bind the COPI coat subunits [34, 65, 66], it will be intriguing to study their involvement in escorting mTORC1 to the lysosomal membrane and the impact on TFEB-mediated lysosomal signaling.

Our dissection of the molecular mechanisms underpinning GOLPH3 interactions with the mTOR pathway now sets the stage to understand the notable effects of GOLPH3 overexpression on mTOR signaling and autophagy observed in a broad set of cancer contexts. It also predicts that the GOLPH3/mTOR/TFEB axis might be altered during neurodegeneration.

## Methods

### Fly stocks and transgenes

For most experiments *Drosophila* strains were cultured in standard cornmeal-agar medium (#789211, NutriFly BF, Genesee Scientific) and maintained at 25 °C. The RNAi experiments were performed at 28 °C as previously described [35]. The *sau*^*z2217*^ mutant strain was described previously [35, 67]. *UAS-dGOLPH3* RNAi (#46150, [35]) and *UAS-Tctp* RNAi (# 26632) were obtained from the Vienna Drosophila Resource Center Collection (VDRC, [44]). The following fly strains were from the Bloomington Drosophila Stock Center (BDSC, Indiana University, Bloomington, IN, USA): *UAS-Rheb* (#9689)*, UAS-GFP* (#4775), *Df(2* *L)Exel7010* (#7782), *en-Gal4* (#30564)*, nub-Gal4* (#86108), *ey-Gal4* (#5534), *elav-Gal4* (#8765), and *bam-Gal4* (#80579, [68]). The following fly strains were obtained from FlyORF (University of Zurich, [69]): *UAS-dGOLPH3-HA* (#F002769)*, UAS-Tctp-HA* (#F002479)*, UAS-14-3-3ζ-HA* (#F001064). *bam-Gal4* and *tub-Gal4/tub-Gal80*^*ts*^ (gift of Dr. Timothy Megraw, Florida State University, USA) were used to deplete dGOLPH3 respectively in spermatocytes and in larval/pupal tissues and to express dGOLPH3-HA, Tctp-HA, and 14-3-3ζ-HA proteins. The fly strains expressing either GFP, or fluorescent tagged-dGOLPH3 were described previously: *GFP* [34], *dGOLPH3-RFP* [34], *GFP-dGOLPH3* [35], and *GFP-dGOLPH3*^*K167A/R170L*^ [35].

### RT-qPCR

20 fly heads per genotype were dissected in cold 1X Dulbecco’s phosphate-buffered saline (PBS, #D8537, Sigma-Aldrich) upon leaving flies at 28 °C for 24 h prior dissection for 1 day for experiments with mutant combinations and 3 days for interference experiments. Fly heads were collected in TRIZOL reagent (#15596-018, Invitrogen) and homogenized using plastic pestles. Quick-RNA Tissue/Insect Microprep Kit (#R2030, Zymo Research) was used for RNA extraction. Retrotranscription was performed using LunaScript® RT SuperMix Kit (#E3010, New England BioLabs) and qPCR was performed using Luna® Universal qPCR Master Mix (#M3003, New England BioLabs). CFX Connect Real Time PCR Detection System (#1855201, Bio-Rad) was used to perform the qPCR reaction. Primer Sequences described previously [50, 51, 70–72] are reported in Table S1.

### S2 cell culture

The S2 cell line was cultured at 25 °C in Schneider’s insect medium (#59895; Sigma-Aldrich) supplemented with 10% heat-inactivated fetal bovine serum (#F9665-500ML; Sigma-Aldrich) and penicillin-streptomycin.

### Protein extracts and western blotting

Protein extracts of adult testes, pupae, adult heads and S2 cells were obtained as described below. S2 cells were homogenized in 1 ml of Lysis buffer [25 mM Tris–HCl pH 7.4; 150 mM NaCl, 1 mM EDTA; 1% NP40] with protease inhibitor cocktail (#11697498001, Roche) and phosphatase inhibitors (#04906845001, Roche). At least 200 testes per each genotype were homogenized in 500 µl of Lysis buffer with protease and phosphatase inhibitor cocktail on ice. To obtain protein extracts from pupae, 20 pupae per genotype were homogenized in 600 µl of Buffer A [20 mM Tris-HCl pH 7.5, 100 mM NaCl, 5 mM MgCl2, 10% Sucrose, 1 mM EDTA, 0,2% NP-40] with protease and phosphatase inhibitors on ice. After filtration with cell strainers, lysates from pupae were clarified by centrifugation at 12,200×*g* for 10 min. To obtain adult head extracts, 10 fly heads per genotype were dissected in cold 1X PBS upon leaving flies at 28 °C prior dissection for 1 day for experiments with mutant combinations and 3 days for RNAi interference experiments. Protein concentration was quantified using the Pierce BCA Protein Assay Kit (#233225, Thermo Scientific). Protein samples were separated on Mini-protean TGX Stain-Free Gel gels (Bio-Rad Laboratories) and blotted to PVDF membranes (#1620177, Bio-Rad) using the Trans-Blot Turbo TM Transfer System (#1704150, Bio-Rad). Membranes were blocked in EveryBlot Blocking Buffer, (#12010020, Bio-Rad) and incubated with antibodies diluted in TBS-T [20 mM Tris-HCl pH 7.5, 150 mM NaCl, 0.05% Tween 20]. Primary antibodies were as follows: rabbit anti-Phospho-*Drosophila* p70 S6 Kinase (1:1000; #9209, Cell Signaling Technology), guinea pig anti-S6 Kinase (1:3000; provided by Prof. A. Teleman, German Cancer Research Center, Heidelberg Germany, [73]), guinea pig anti-Rheb (1:1000; Prof. A. Teleman, [74]), rabbit anti-Tctp (1:1000; gift from Dr. Dae-wook Yang and Prof. Kwang-Wook Choi, Korea Advanced Institute of Science and Technology, Daejeon, Korea, [22]), rabbit anti-GAPDH (1:5000, #GTX100118, GeneTex), rabbit monoclonal anti-HA (1:1000; #3724, Cell Signaling Technology), mouse monoclonal anti-RFP (1:1000; # 6G6, Chromotek), rabbit anti-GFP (1:2500; TP-401, Torrey Pines Biolabs), mouse anti-dGOLPH3 (1:2500; #S11047/1/56, [35]), rabbit anti-dGOLPH3 (1:2500; #L11047/G49139/77, [35]) mouse anti-6X-His tag (1:1000, #MA1-21315, Invitrogen), rabbit anti-Atg8a (1:5000; #109364, Abcam), rabbit anti-ref(2)P (1:1000; #178440, Abcam), mouse anti-actin (1:10000; #JLA20 concentrated form, Developmental Studies Hybridoma Bank). HRP-conjugated secondary antibodies were as follows: goat anti-rabbit IgG (H + L) (1:5000; #170-6515, Bio-Rad), goat anti-mouse IgG (H + L) (1:5000; #170-6516, Bio-Rad), and goat anti guinea-pig IgG (1:2000, #AP108P, Sigma-Aldrich). Membranes were incubated 5 min with ECL substrate (#1705062 and #1705060, Bio-Rad) and the HRP-ECL reaction was revealed using the ChemiDoc^TM^ XRS gel imaging system (Bio-Rad). Band intensity quantification was performed using the gel analyzer tool in Fiji/ImageJ software [75].

### Co-IP analysis

Co-IP experiments were performed from lysates of pupae expressing GFP/RFP tagged dGOLPH3 or HA-tagged Tctp. Co-IP from lysates expressing either GFP or RFP tagged dGOLPH3 were performed using GFP/RFP trap-A purchased from ChromoTek (#gta-20, #rta-20). IP of Tctp-HA was performed using monoclonal anti-HA-Agarose beads (#A2095-1ml, Sigma-Aldrich), following the protocol described previously [34]. Lysates from Or-R pupae or pupae expressing tagged proteins, were precleared by incubation for 1 h on wheel at 4 °C with control agarose beads (bab-20, ChromoTek). 4% of each lysate was retained as the “input”. The beads were rinsed once with ice cold Buffer A [20 mM Tris-HCl pH 7.5, 100 mM NaCl, 5 mM MgCl2, 10% Sucrose, 1 mM EDTA, 0,2% NP-40] and washed extensively (4×5 min) on the wheel at 4 °C. For the elution of GFP/RFP tagged dGOLPH3 and its molecular interactors, after the final wash, beads were resuspended in 30 µL of SDS sample buffer [20% glycerol, 4% SDS, 0.2% BBF, 100 mM Tris-HCl (pH 6.8), 200 mM DTT] and boiled for 10 min. The Tctp-HA protein complex was eluted from the beads by adding 50 µl of 200 mM glycine pH 2.0 (1:1). The step was repeated two times and the eluate was neutralized by adding an equal volume of 1 M Tris pH 8.0. Finally, proteins were precipitated with 4 volumes of ice-cold acetone. Co-IP experiments were performed in triplicate.

### GST pull-down and in vitro pull-down assays

To generate the GST tagged proteins used in the GST pull-down, the full-length cDNAs corresponding to *Tctp, Lst8* and *14-3-3ζ* were cloned into pGEX-6p-2 (#27-4598-01, GE Healthcare Life Sciences). GST, GST-14-3-3ζ and GST-Tctp proteins were expressed in BL21-Codon Plus cells (#230245; Stratagene). GST-Lst8 was expressed in ActicExpress (DE3) cells (#230192, Agilent Technologies). Proteins were purified using glutathione–Sepharose 4B beads (#17-0756-01, GE Healthcare) as described previously [36]. GST pull-down experiments were performed with lysates from *Drosophila* S2 cells or *Drosophila* tissues. Lysates were incubated with either GST or the GST–tagged protein (4 µg) bound to glutathione–Sepharose 4B beads, with gentle rotation, at 4 °C for 2 h. After rinsing in wash buffer (25 mM Tris-HCl pH 7.4, 150 mM NaCl, 1% NP-40, 1 mM EDTA, protease and phosphatase inhibitors) three times, the beads were boiled in SDS sample buffer and separated by SDS-PAGE. Bound proteins were analyzed by WB as described above. Before immunoblotting, PVDF membranes were stained with Ponceau (#P3504, Sigma-Aldrich). Direct interaction assays between 6XHis-dGOLPH3 [36] and either GST-Tctp or GST-Lst8 proteins, were performed using the protocol described previously [36]. Briefly, 6XHis-dGOLPH3 was incubated with GST or GST-tagged proteins bound to glutathione–Sepharose 4B beads for 1 h on wheel at 4 °C. The beads were washed extensively (4 × 5 min) at 4 °C with ice cold wash buffer (25 mM Tris-HCl pH 7.4, 150 mM NaCl, 1% NP-40, 1 mM EDTA, Protease and phosphatase inhibitors). 6XHis-dGOLPH3 bound to each protein was detected by WB. GST pull-down experiments were performed in triplicate.

### Yeast two-hybrid assay

The assay was performed as described previously [76] using the B42/lexA system with EGY48 (*Mata his3 ura3 trp1 6lexAOP-LEU2*; *lexAOP-lacZ* reporter on plasmid pSH18-34) as the host strain [77]. Full-length cDNAs corresponding to *Tctp, Lst8* and *14-3-3ζ* were cloned into pJG4-5 (prey) whereas the cDNA of *dGOLPH3* was used as a bait and cloned into the pEG202 vector, as described in [36]. The yeast strain was co-transformed with various combinations of bait (pEG202) and prey (pJG4-5) plasmids carrying *dGOLPH3*, *Tctp*, *Lst8, 14-3-3ζ* cDNAs. Detailed information on the yeast strains can be found in Table S2. To test for two-hybrid interaction, yeast strains were spotted on 5-bromo-4-chloro-3-indolyl-β-d-galactopyranoside (X-GAL, #B4252, Sigma-Aldrich) selective synthetic plates containing either raffinose (RAFF, prey not induced, #R7630, Sigma-Aldrich) or 2% galactose (GAL, prey expressed, #G5388, Sigma-Aldrich), as described previously [77]. The yeast two-hybrid results, were quantified by using the β-galactosidase assay [76]. Briefly, after growing yeast strains in a selective medium containing galactose, protein extracts were prepared to test β-galactosidase enzyme activity using *o*-nitrophenyl-d-galactoside (ONPG, #N1127, Sigma-Aldrich) as a substrate. The assays were performed in triplicate to calculate average β-galactosidase units and SEM.

### Microscopy and histology

Adult wings were dissected in isopropanol and mounted using the Canada balsam (#C1795, Sigma-Aldrich). Images of wings and eyes were captured using ZEISS Axiocam 105 color camera. Images of ommatidia and wing hairs were taken using an Eclipse Nikon Axioplan epifluorescence microscope, with a Plan Apo 10x/NA = 0.25 objective lens and a QICAM Fast 1394, Cooled charge-coupled device. Wing, eye and ommatidia areas were measured from images with Fiji/ImageJ software using the freehand selection tool; whereas wing lengths were measured using the straight lines tools between the two most distal points of the wing. The number of wing hairs on the dorsal wing surface was counted using the Analyse Particles tools of Fiji/ImageJ, applied to a 22,500 μm^2^ area, selected just posterior to the posterior crossvein (one wing hair corresponds to one cell). Immunofluorescence analysis of primary spermatocytes were made with testes from third instar larvae. Testes were dissected in PBS and then transferred into 5 µl of 4% methanol-free formaldehyde (#18814-20, Polysciences) in PBS on a 20 × 20 coverslip. After 1 min, samples were gently squashed, left for 6 min at room temperature and then immersed into liquid nitrogen. After coverslip removal with a razor blade, preparations were rinsed 2×5 min in PBS. Samples were permeabilized (2 × 10 min) in PBS with 0.1% Triton X-100 (PBT) and then blocked with 3% BSA in PBT for 30 min. Primary antibodies were as follows: guinea pig anti-Rheb (1:1000; Prof. A. Teleman, [74]) and rabbit anti-Lava lamp (1:300; gift from O. Papoulas, University of Texas at Austin; [46]). Secondary antibodies were: Alexa Fluor 488 AffiniPure F(ab’)_2_ Fragment Goat Anti-Guinea Pig IgG (H + L) (#106-546-003, Jackson Immunoresearch) and Alexa 555-conjugated goat anti-rabbit IgG (1:300, #A21430, Life Technologies). Samples were mounted in Vectashield Vibrance with DAPI (#H-1800, Vector Laboratories). Fluorescence images from fixed spermatocytes were acquired using a Zeiss Axio Observer Z1 (Carl Zeiss) equipped with a 100x/1.3 NA oil immersion objective and a charged-coupled device (Axiocam 503 mono CCD camera) and an HXP 120 V inclusive built-in power supply, lamp module. Images were processed using the ZEN2 software and Adobe Photoshop. Fluorescence intensity measurements of Rheb protein at the Golgi were made from images using Fiji/ImageJ software as described previously [36]. The Golgi compartment was demarcated using the freehand selection tool and the mean signal intensity of the Rheb protein was measured in the selected area. Fluorescence and DIC Images of living spermatocytes expressing fluorescence-tagged-dGOLPH3 were captured using the Zeiss Cell Observer Z1 microscope (see above for the details) equipped with a 63×/1.4 objective.

### Statistical analysis

The statistical analysis on fly lethality and rescue experiments was performed using the Fisher’s exact test (Graphpad Prism). For all the other experiments, data represent the mean ± standard deviation (SD) or standard error of the mean (SEM) from three independent experiments. Statistical significance was performed using the Unpaired two-tailed Student’s *t* test using Prism 8 (Graphpad Prism, USA). Statistical parameters of individual experiments (value of n, mean, SEM, *p* value) are reported in each figure legend in the paper.

## Supplementary information

Supplementary Information

Original Data File

checklist

## Data Availability

All data reported in this paper will be shared upon request.
