# Supplementary material for: GOLPH3 protein controls organ growth by interacting with TOR signaling proteins in Drosophila
Source: Cell Death Dis. 2022 Nov 27;13(11):1003. doi: 10.1038/s41419-022-05438-9 (PMC9701223; doi:10.1038/s41419-022-05438-9)
Supplement: Supplementary file 1 — Supplementary Information [file 41419_2022_5438_MOESM1_ESM.pdf]

Supplementary Information

SUPPLEMENTARY FIGURES

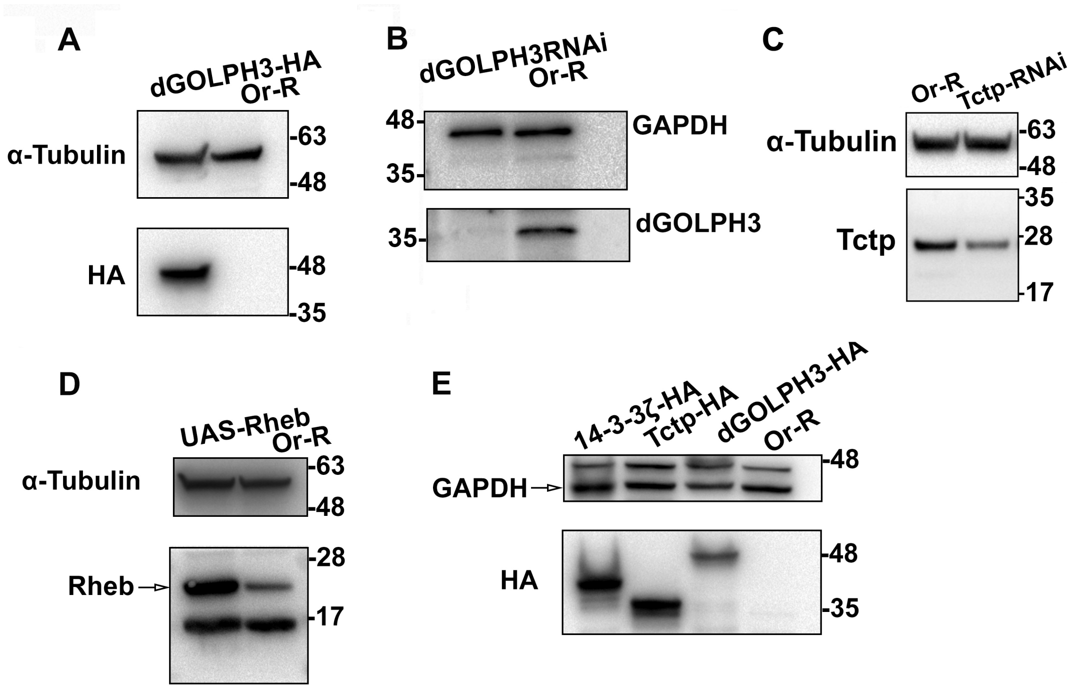

Figure S1. Western blotting analysis to test protein expression in larval extracts from animals carrying the indicated transgenes

The *tub-Gal4/tub-Gal80^{ts}* system, upon a shift to a higher temperature (29°C), was used to induce UAS-GOLPH3-HA transgene (A), to drive UAS-*dGOLPH3*RNAi (B) and UAS-*Tctp* RNAi (C) and to induce the expression of the transgenes UAS-Rheb (D), UAS-Tctp-HA (E) and UAS-14-3-3z-HA (E). Tubulin or GAPDH were used as a loading control. Molecular masses are in kilodaltons.

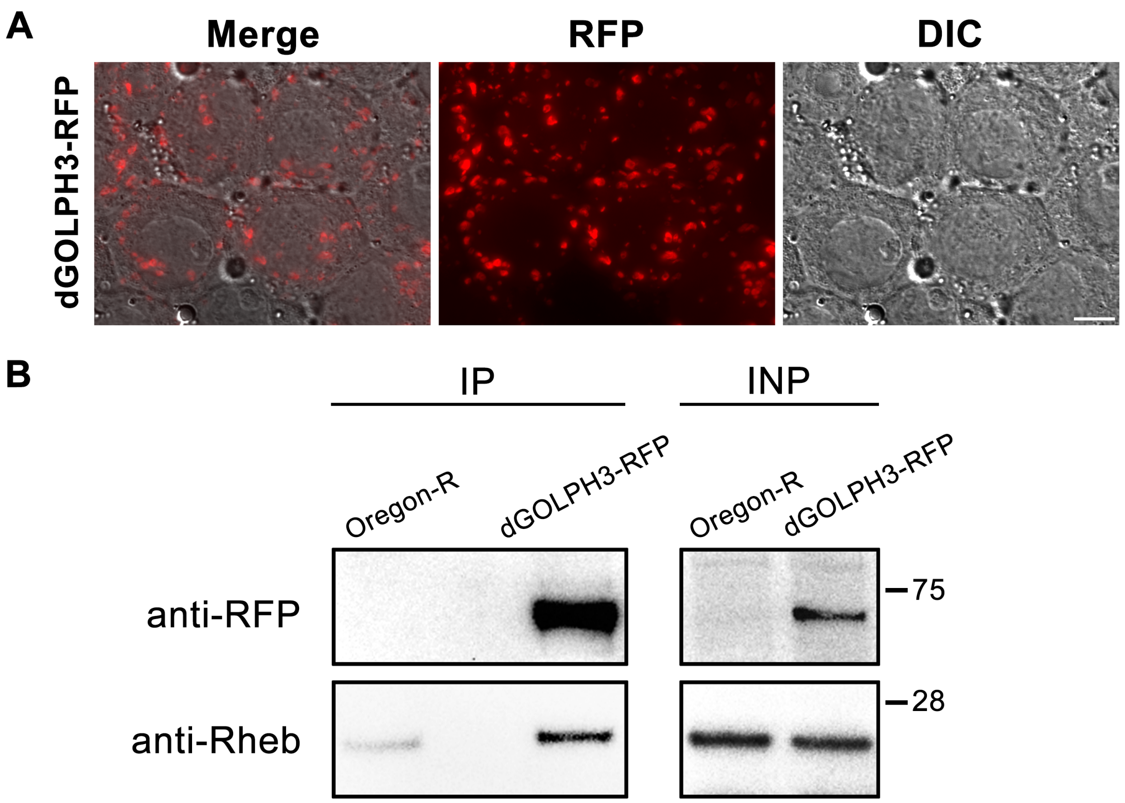

Figure S2. RFP tagged dGOLPH3 protein localizes to the Golgi and interacts with Rheb

(A) Fluorescence and corresponding DIC micrographs of live wild type spermatocytes expressing dGOLPH3-RFP during prophase. Scale bar, 10 µm. (B) Co-IP analysis from protein extracts of wild type pupae (Oregon-R) or pupae expressing dGOLPH3-RFP. Protein extracts were immunoprecipitated with RFP trap and blotted to detect either Rheb or dGOLPH-RFP. 2% of the input and 50% of the immunoprecipitates were loaded and probed with indicated antibodies. Molecular masses are in kilodaltons.

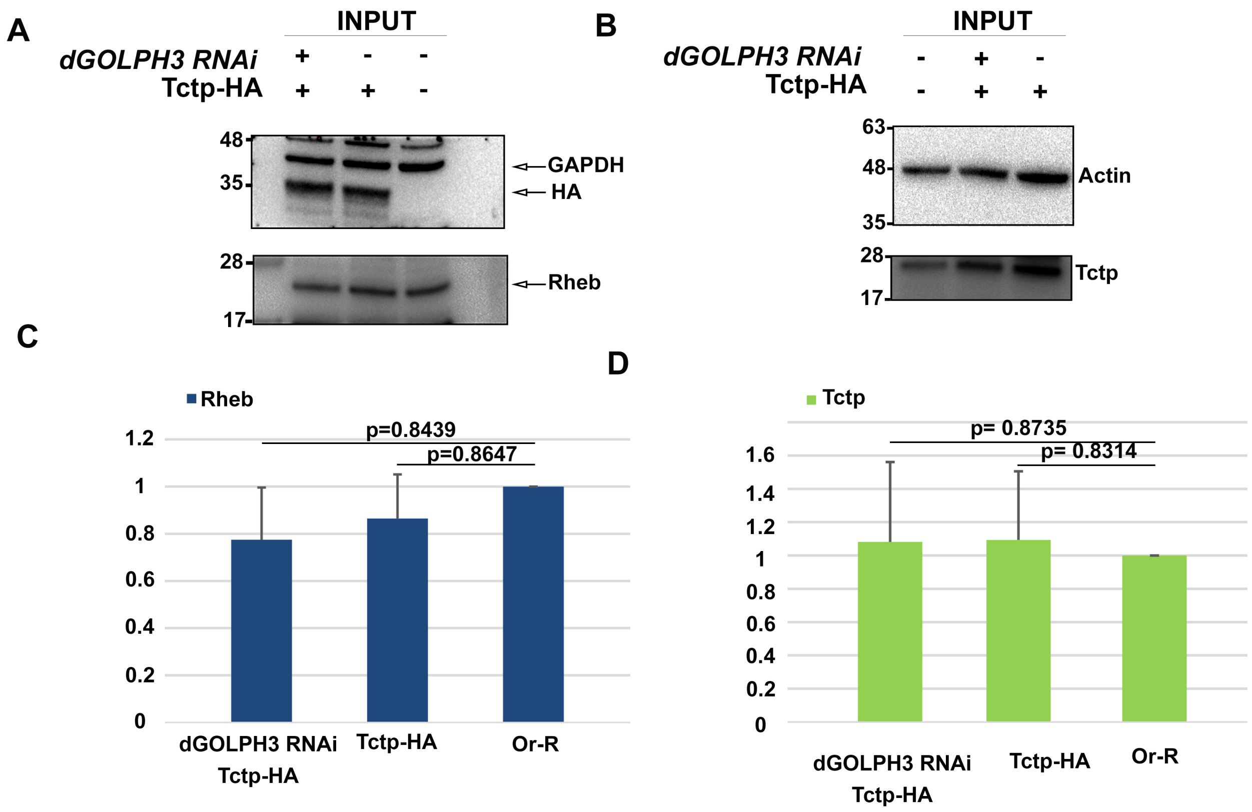

Figure S3. Levels of endogenous Tctp and Rheb proteins are not noticeably affected by dGOLPH3 knockdown and Tctp-HA expression

Western blot from protein extracts of *Drosophila* pupae to test the effects of *dGOLPH3* RNAi and Tctp-HA on the levels of Rheb (A,C) and endogenous Tctp (B,D). GAPDH (A) and Actin (B) were used as loading control. Molecular masses are in kilodaltons. (C,D) Quantification of the expression levels of Rheb (C) and Tctp (D) proteins in western blots shown respectively in A and in B. Band intensities are from three independent experiments. The intensity of each band relative to the intensity of loading control was normalized to the wild-type control (Or-R). Error bars, SD, unpaired t-test. Related to Figure 5E.

SUPPLEMENTARY TABLES

Table S1. Primer Sequences

**Primer**	**Sequence**	**Ref.**
Rp49 FOR	5'ACGTTGTGCACCAGGAACTT3'	70
Rp49 REV	5'TACAGGCCCAAGATCGTGAA3'	70
Mitf FOR	5'GCGTTCTTCTTCAGGGATTG3'	50
Mitf REV	5'ACTTACGCTCGGCGAAATAG3'	50
Atg8a FOR	5'CATGGGCTCCCTGTACCA3'	71
Atg8a REV	5'CTCATCGGAGTAGGCAATGT3'	71
ref(2)P FOR	5'ATGCCGGAGAAGCTGTTGAA3'	72
ref(2)P REV	5'ATCAGCGTCGATCCAGAAGG3'	72
VhaPPA1-1 FOR	5'ATCTTCGGTTCGGCCATC3'	71
VhaPPA1-1 REV	5'ATAATGGAGTGGCGAAGGAC3'	71
Vha16-1 FOR	5'CACAACAACAACAGATAGACAAACG3'	51
Vha16-1 REV	5'GAAGCTGCTGCTGATGTTGAT3'	51
Vha44 FOR	5'TTGGTTCGTTGGCTGAAGGT3'	71
Vha44 REV	5'GCACGGATTCCACAAACACG3'	71
Vha100-1 FOR	5'AATCCCGATGTCAACGCTTTC3'	71
Vha100-1 REV	5' TTCTCCAGGTAACGCAGCTTG3'	71
Vha14-1 FOR	5'CCAGCTGACCCGTACATTT3'	71
Vha14-1 REV	5'CCAGCTGACCCGTACATTT3'	71

Table S2. Yeast strains used in this study

**Name**	**Genotype**	**Source**
RF1958	*Mat a, his3, ura3, trp1, 6lexAOP-LEU2; leXAOP-lacZ* reporter on plasmid pSH18-34, Golph3 cloned into pEG202, PJG4-5	Sechi et al., 2020
RF2200	*Mat a, his3, ura3, trp1, 6lexAOP-LEU2; leXAOP-lacZ* reporter on plasmid pSH18-34, Golph3 cloned into pEG202, LST8 cloned into PJG4-5	this study
RF2201	*Mat a, his3, ura3, trp1, 6lexAOP-LEU2; leXAOP-lacZ* reporter on plasmid pSH18-34, Golph3 cloned into pEG202, 14-3-3 cloned into PJG 4-5	this study
RF2202	*Mat a, his3, ura3, trp1, 6lexAOP-LEU2; leXAOP-lacZ* reporter on plasmid pSH18-34, pEG202, LST8 cloned into PJG4-5	this study
RF2203	*Mat a, his3, ura3, trp1, 6lexAOP-LEU2; leXAOP-lacZ* reporter on plasmid pSH18-34, pEG202, 14-3-3 cloned into PJG4-5	this study
RF2210	*Mat a, his3, ura3, trp1, 6lexAOP-LEU2; leXAOP-lacZ* reporter on plasmid pSH18-34 (URA3), Golph3 cloned into pEG202 (HIS3, 2 micron), TCTP cloned into PJG4-5 (TRP1, 2 micron)	this study
RF2211	*Mat a, his3, ura3, trp1, 6lexAOP-LEU2; leXAOP-lacZ* reporter on plasmid pSH18-34 (URA3), pEG202 (HIS3, 2 micron), TCTP cloned into PJG4-5 (TRP1, 2 micron)	this study
