# Supplementary material for: GOLPH3 protein controls organ growth by interacting with TOR signaling proteins in Drosophila
Source: Cell Death Dis. 2022 Nov 27;13(11):1003. doi: 10.1038/s41419-022-05438-9 (PMC9701223; doi:10.1038/s41419-022-05438-9)

Figure 1A

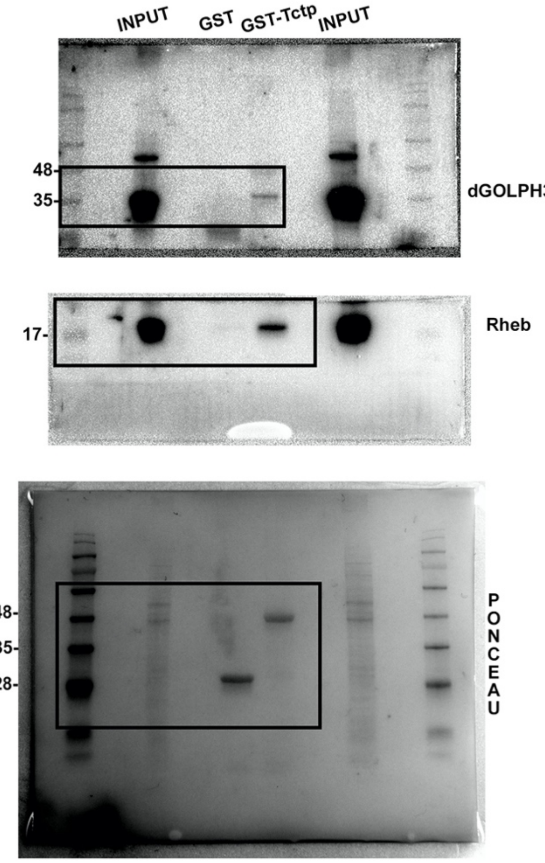

Figure 1B

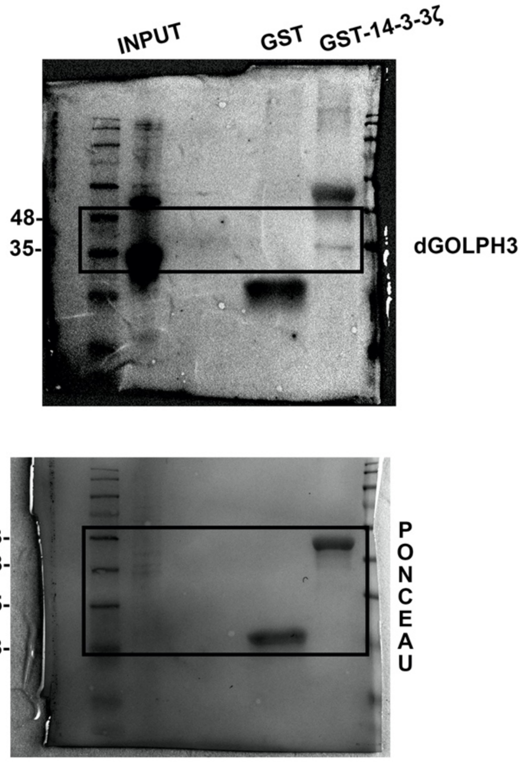

Figure 1C

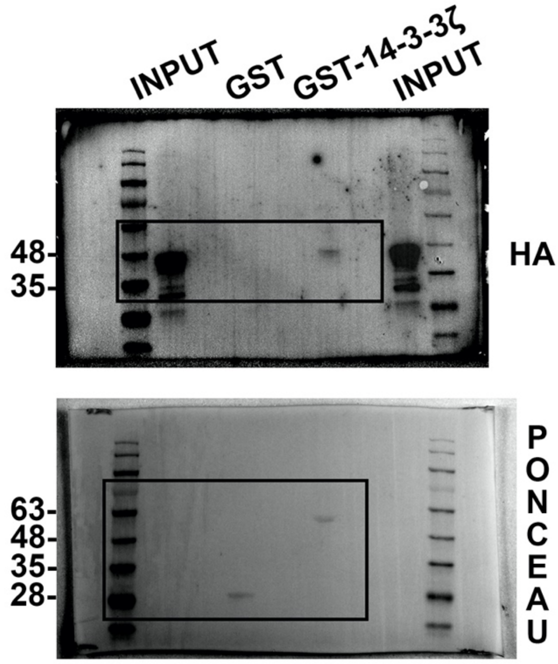

Figure 1D

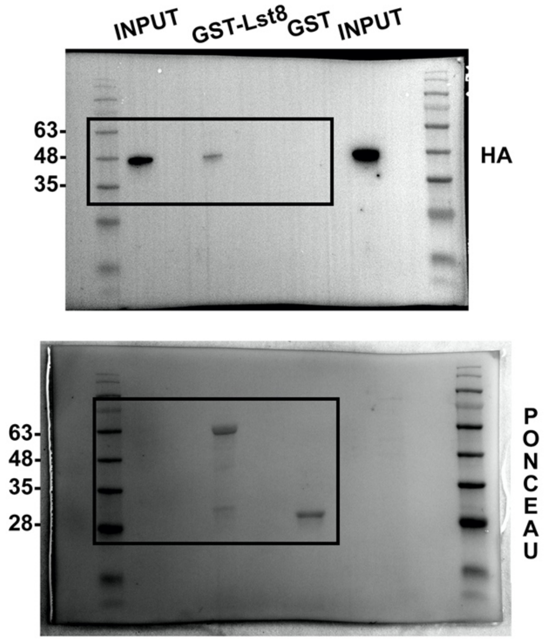

Figure 1E

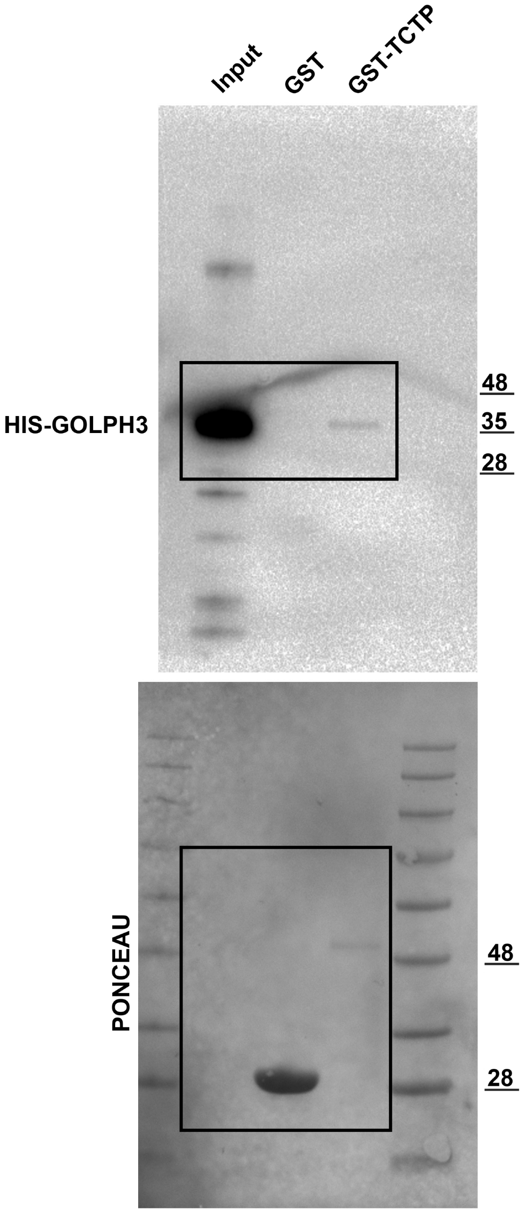

Figure 1F

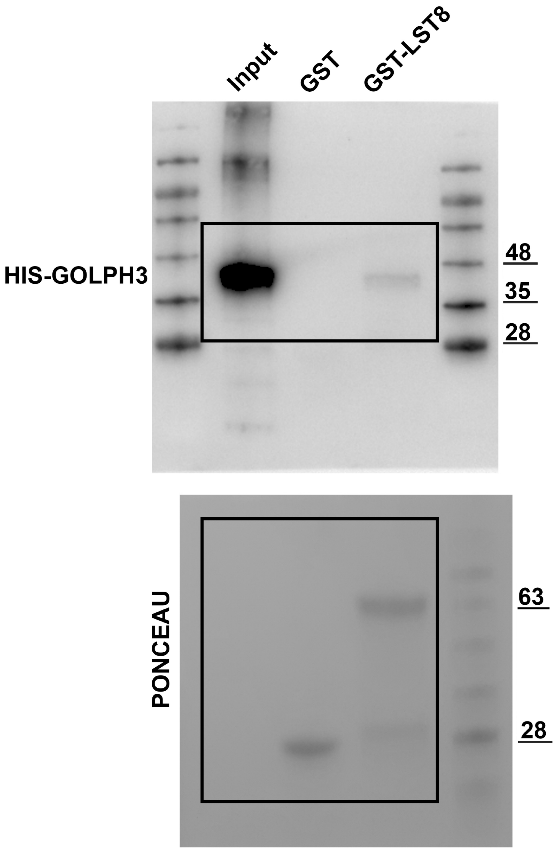

Figure 5D

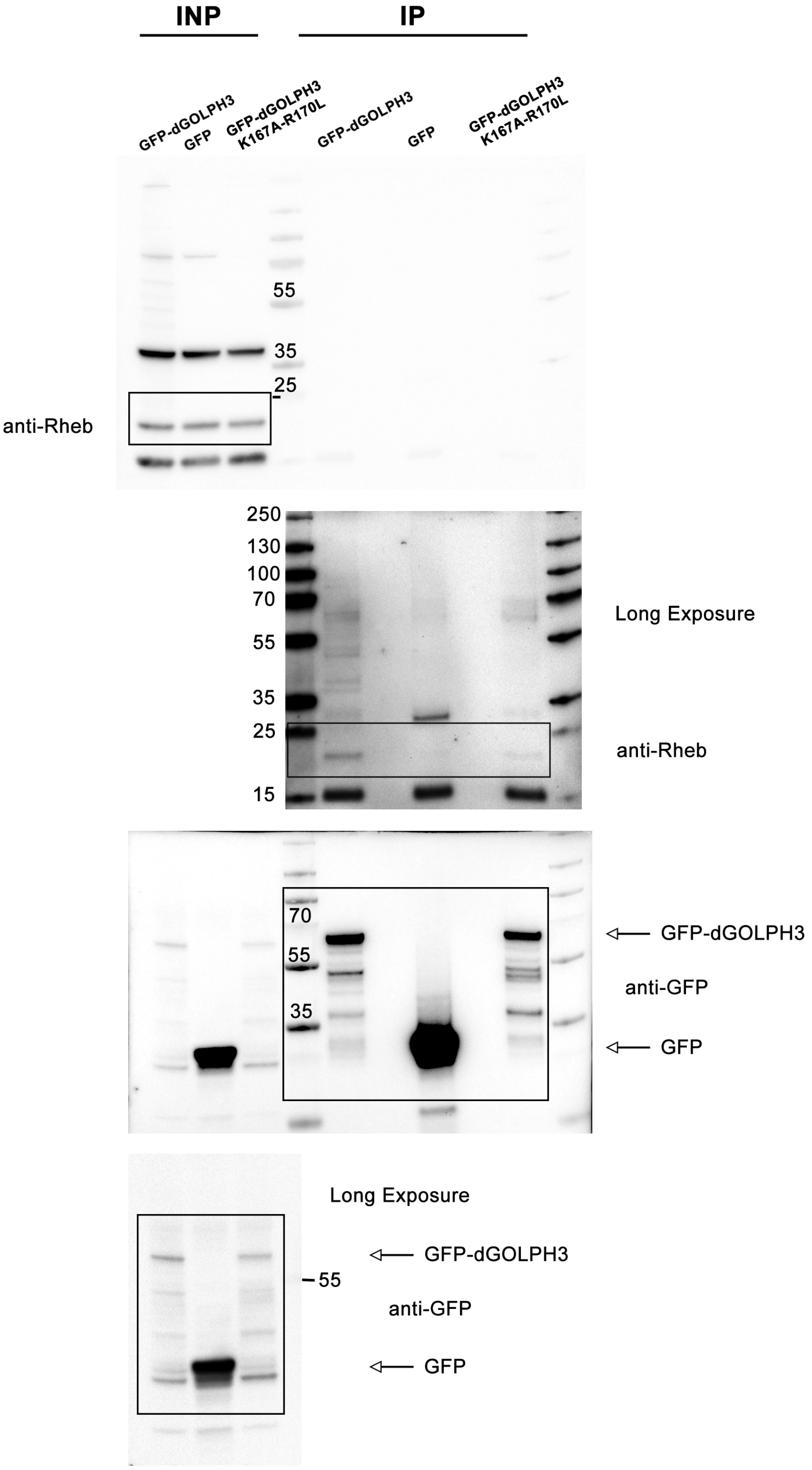
INP
IP
GFP-dGOLPH3
GFP
GFP-dGOLPH3 K167A-R170L
GFP-dGOLPH3
GFP
GFP-dGOLPH3 K167A-R170L
55
35
25
anti-Rheb
250
130
100
70
55
35
25
15
Long Exposure
anti-Rheb
70
55
35
GFP-dGOLPH3
anti-GFP
GFP
Long Exposure
GFP-dGOLPH3
55
anti-GFP
GFP

Figure 5E

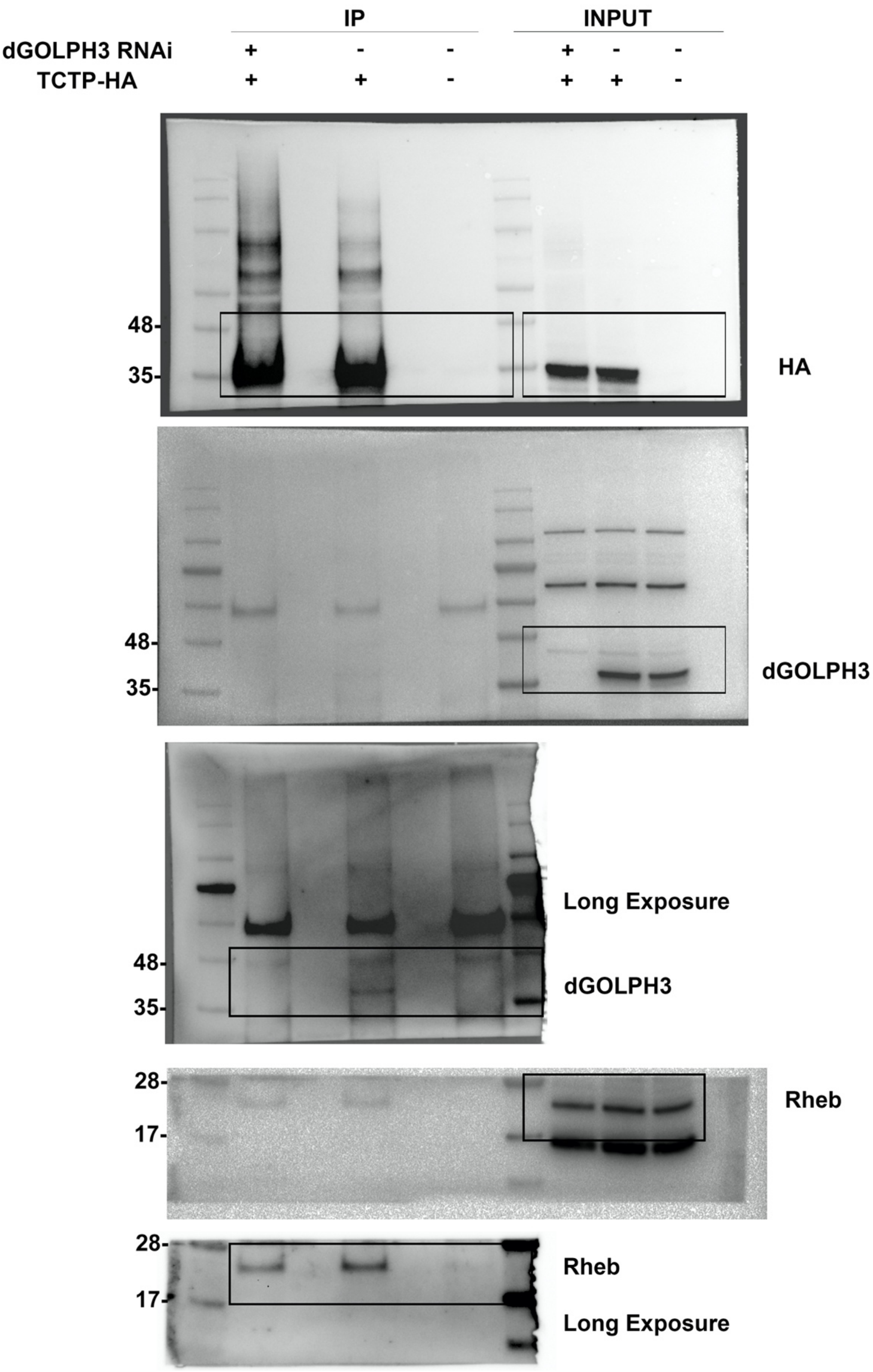
IP
INPUT
dGOLPH3 RNAi
TCTP-HA
+ - - + - -
+ + - + + -
48-
35-
HA
48-
35-
dGOLPH3
48-
35-
Long Exposure
dGOLPH3
28-
17-
Rheb
28-
17-
Rheb
Long Exposure

Figure 6A

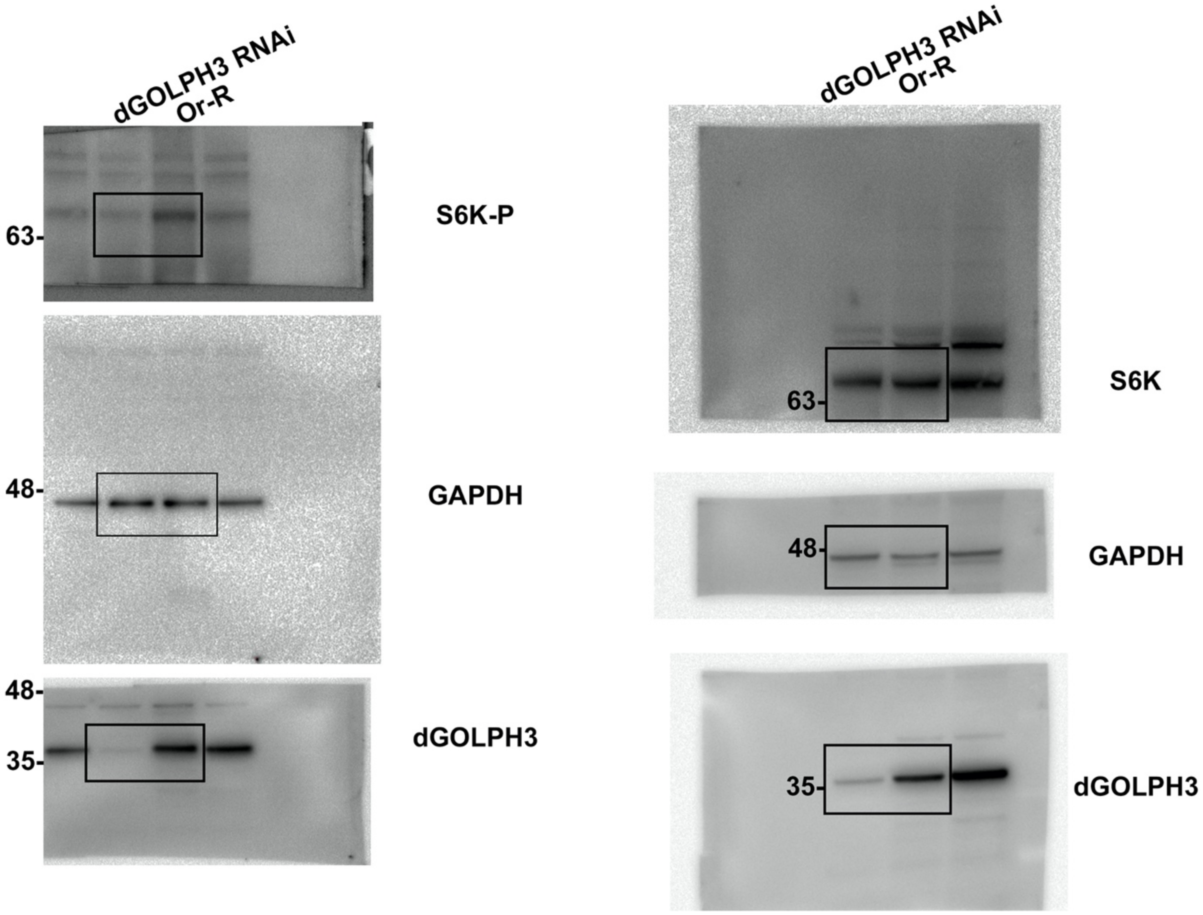

Figure 6C

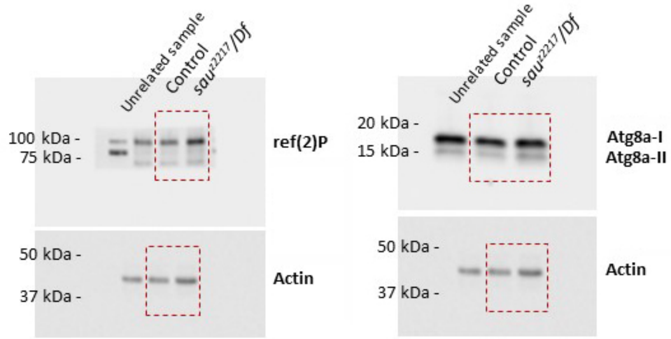

Figure 6F

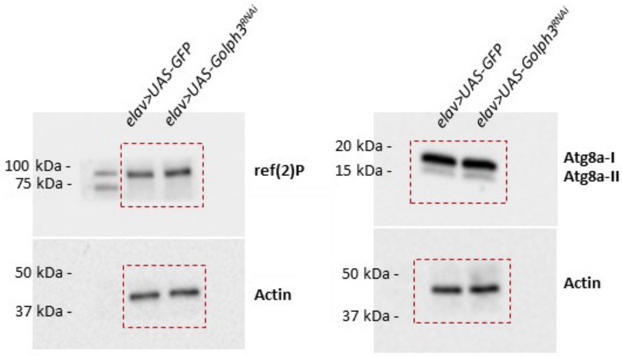

Figure S1A

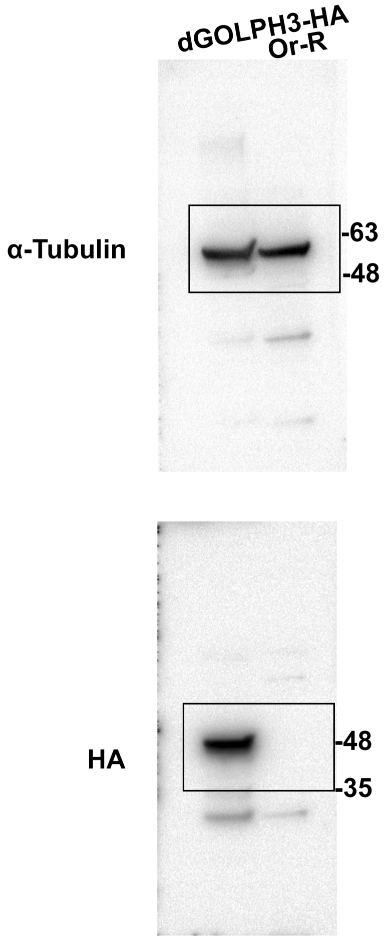

Figure S1B

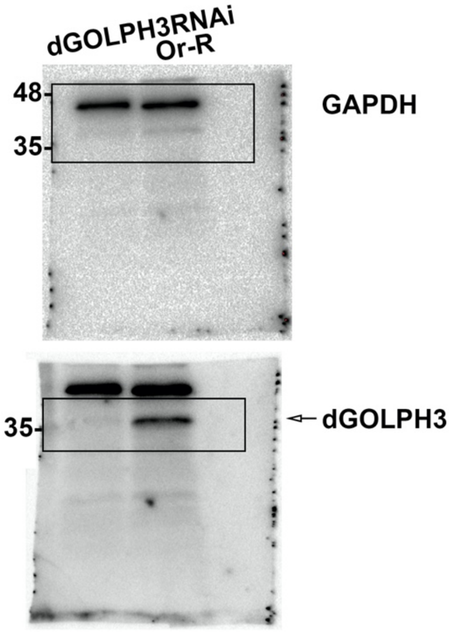

Figure S1C

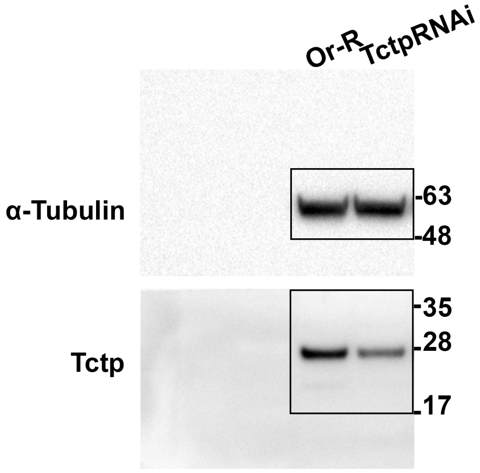

Figure S1D

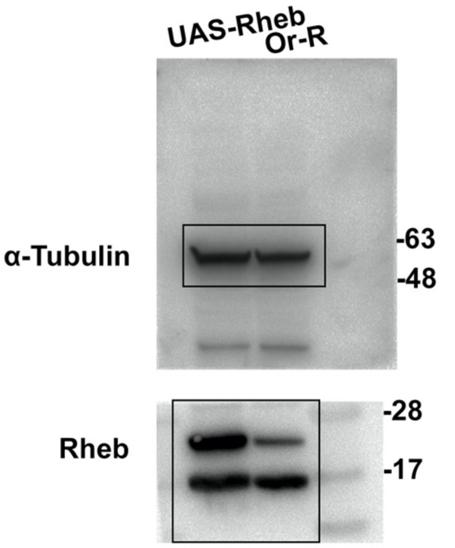

Figure S1E

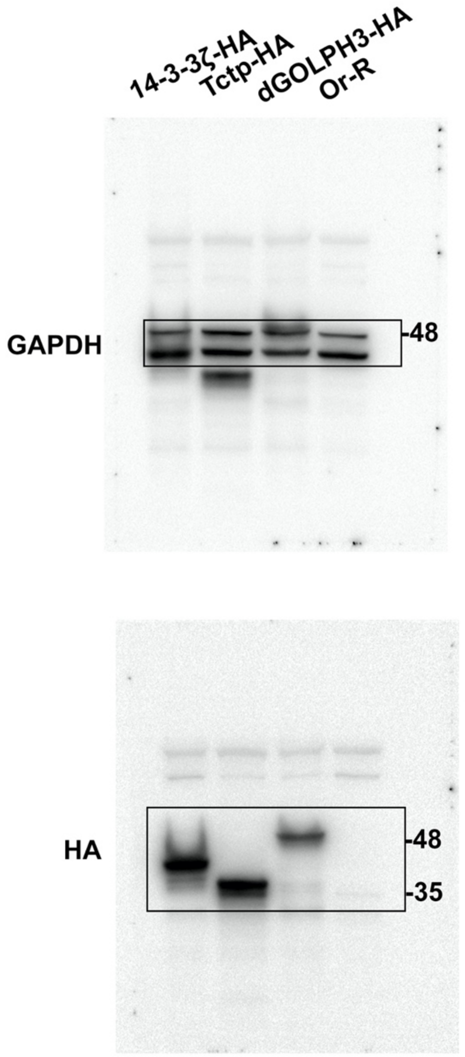

Figure S2B

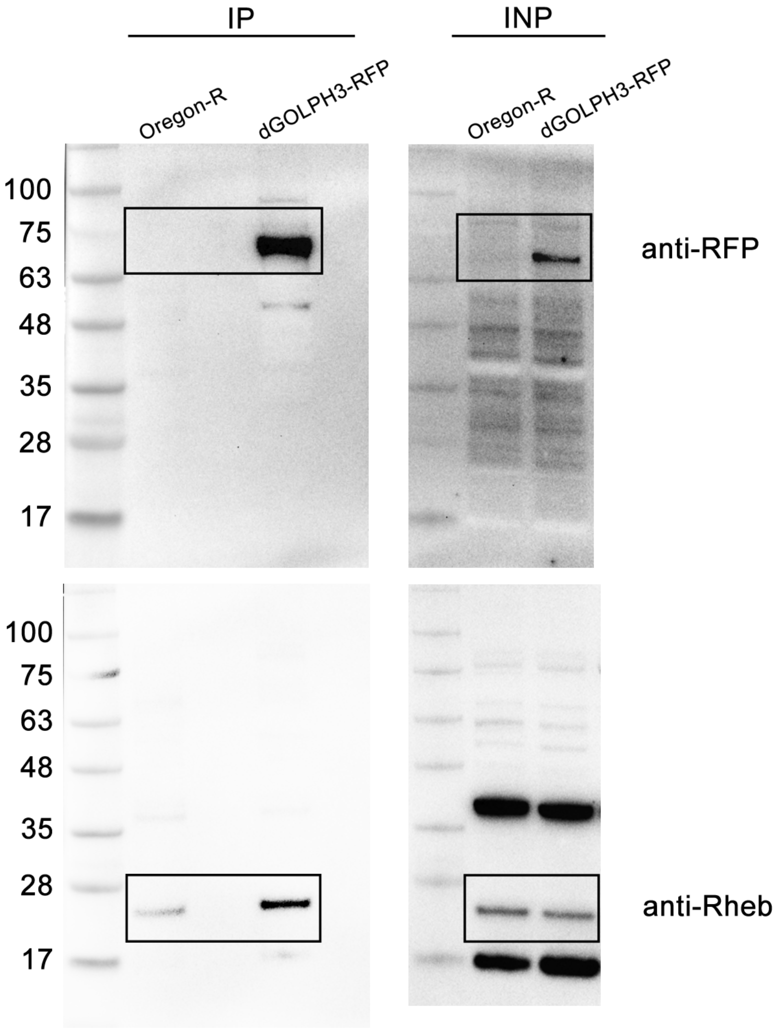

IP
INP
Oregon-R
dGOLPH3-RFP
Oregon-R
dGOLPH3-RFP
100
75
63
48
35
28
17
anti-RFP
100
75
63
48
35
28
17
anti-Rheb

Figure S3A

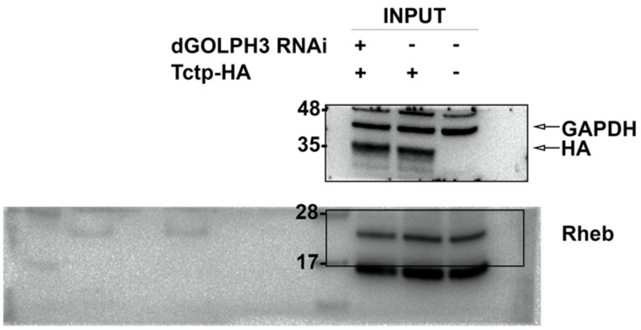

Note that the membrane incubated for Rheb and HA (Figure 5E), has been incubated for GAPDH as loading control

Figure S3B

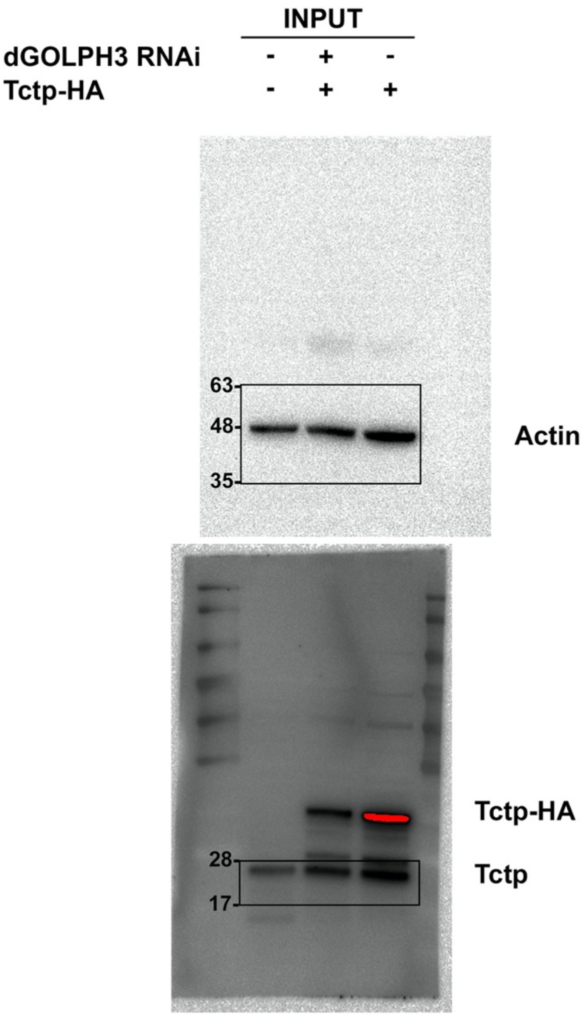

Supplement: Supplementary file 2 — Original Data File [file 41419_2022_5438_MOESM2_ESM.pdf]
